# Significance of Microbiota in Obesity and Metabolic Diseases and the Modulatory Potential by Medicinal Plant and Food Ingredients

**DOI:** 10.3389/fphar.2017.00387

**Published:** 2017-06-30

**Authors:** Hoda M. Eid, Michelle L. Wright, N. V. Anil Kumar, Abdel Qawasmeh, Sherif T. S. Hassan, Andrei Mocan, Seyed M. Nabavi, Luca Rastrelli, Atanas G. Atanasov, Pierre S. Haddad

**Affiliations:** ^1^Natural Health Products and Metabolic Diseases Laboratory, Department of Pharmacology and Physiology, Université de MontréalMontréal, QC, Canada; ^2^Canadian Institutes of Health Research Team in Aboriginal Antidiabetic MedicinesMontréal, QC, Canada; ^3^Department of Pharmacognosy, University of Beni-SuefBeni-Suef, Egypt; ^4^Nell Hodgson Woodruff School of Nursing, Emory UniversityAtlanta, GA, United States; ^5^Department of Chemistry, Manipal Institute of Technology, Manipal UniversityManipal, India; ^6^Faculty of Pharmacy, Hebron UniversityHebron, Palestine; ^7^Department of Natural Drugs, Faculty of Pharmacy, University of Veterinary and Pharmaceutical Sciences BrnoBrno, Czechia; ^8^Department of Pharmaceutical Botany, Iuliu Hatieganu University of Medicine and PharmacyCluj-Napoca, Romania; ^9^ICHAT and Institute for Life Sciences, University of Agricultural Sciences and Veterinary MedicineCluj-Napoca, Romania; ^10^Applied Biotechnology Research Center, Baqiyatallah University of Medical SciencesTehran, Iran; ^11^Dipartimento di Farmacia, University of SalernoFisciano, Italy; ^12^Institute of Genetics and Animal Breeding, Polish Academy of SciencesJastrzebiec, Poland; ^13^Department of Pharmacognosy, University of ViennaVienna, Austria; ^14^Department of Vascular Biology and Thrombosis Research, Center for Physiology and Pharmacology, Medical University of ViennaVienna, Austria

**Keywords:** microbiota, natural products, food ingredients, obesity, metabolic diseases

## Abstract

Metabolic syndrome is a cluster of three or more metabolic disorders including insulin resistance, obesity, and hyperlipidemia. Obesity has become the epidemic of the twenty-first century with more than 1.6 billion overweight adults. Due to the strong connection between obesity and type 2 diabetes, obesity has received wide attention with subsequent coining of the term “diabesity.” Recent studies have identified unique contributions of the immensely diverse gut microbiota in the pathogenesis of obesity and diabetes. Several mechanisms have been proposed including altered glucose and fatty acid metabolism, hepatic fatty acid storage, and modulation of glucagon-like peptide (GLP)-1. Importantly, the relationship between unhealthy diet and a modified gut microbiota composition observed in diabetic or obese subjects has been recognized. Similarly, the role of diet rich in polyphenols and plant polysaccharides in modulating gut bacteria and its impact on diabetes and obesity have been the subject of investigation by several research groups. Gut microbiota are also responsible for the extensive metabolism of polyphenols thus modulating their biological activities. The aim of this review is to shed light on the composition of gut microbes, their health importance and how they can contribute to diseases as well as their modulation by polyphenols and polysaccharides to control obesity and diabetes. In addition, the role of microbiota in improving the oral bioavailability of polyphenols and hence in shaping their antidiabetic and antiobesity activities will be discussed.

## Definition of human microbiota

**Figure 1:**
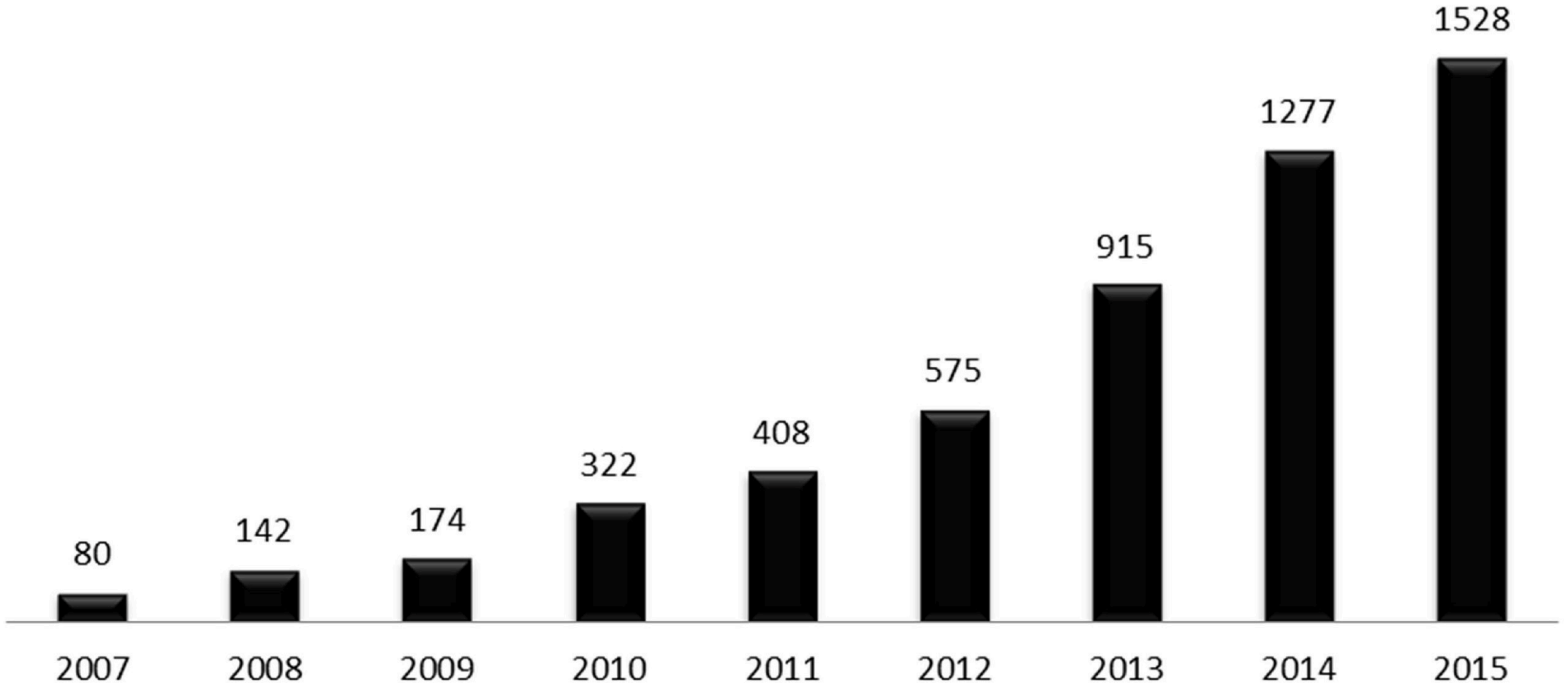
Number of publications related to the human gut microbiota in the last decade, per year. Data were obtained by searching Pubmed (http://www.ncbi.nlm.nih.gov/pubmed/) database using the term “human gut microbiota” on 4th November 2016.

The human microbiota (microflora) refers to an aggregation of a mixture of microorganisms (e.g., bacteria, fungi, archaea, and viruses) that live in human tissues such as skin, uterus, lungs, and in the gastrointestinal tract without causing pathological responses in the host (Hugon et al., 2016). Instead these organisms form a symbiotic relationship with their host. In this relationship, the host provides a shelter and nutrition for the microbes, while the microbes provide the host with essential metabolites such as vitamin K, thiamine, biotin, folic acid, and vitamin B12, digesting polysaccharides into simpler molecules, boosting the immunity to combat pathogens, and competing with the latter for survival (Santacruz et al., 2010; D'Aimmo et al., 2012; LeBlanc et al., 2013). Beyond their traditional benefits, recent studies have implicated microbes residing human gut in modulating brain development (Wang and Wang, 2016), altering metabolic functions (Martinez et al., 2016), hormones and neurochemical production (Baothman et al., 2016). Some 30 years ago, human gut microbiota was largely ignored as a causative factor for illnesses and as a target for pharmacological intervention. Scientists were focused mainly on finding a novel pathogenic bacteria residing in the gut as an underlying cause of illnesses and as a target for drug development (Eggerth and Gagnon, 1933; Dalton, 1951; Walther and Millwood, 1951). In recent years, there has been an increasing trend in this area of research, and the focus has been shifted toward identifying microbial composition of the human gut microbiota (Figure 1), the factors altering this composition, and relating it to the pathogenesis of some diseases such as diabetes (Yamaguchi et al., 2016), autism (Berding and Donovan, 2016), obesity (Valsecchi et al., 2016), and other disorders (Gondalia et al., 2012; Baothman et al., 2016). In this review, we discuss the current advances in human gut microbiota, specifically their identity and diversity within the gastrointestinal tract of healthy adults as well as their contribution to metabolic diseases. We also review current knowledge about the effects of polyphenols and polysaccharides on gut microbiota and their role in controlling obesity and diabetes.

## Identity of microbes colonizing human gut

Microbes (bacteria, archaea, fungi, virus) colonizing human gut establish a complex ecosystem with current evidences confirming large diversity in their number and identity throughout the whole gastrointestinal tract (Hugon et al., 2016). It is now accepted that each individual hold a unique set of microbes (Callaway, 2015) whose composition is highly affected by many factors such as ethnicity, age, environment, and diet (Ursell et al., 2012; Yatsunenko et al., 2012). The exact composition of gut microbiota seems far from being fully identified in view of fast advances in “OMICS” techniques as well as improved culture conditions allowing for identification of new sets of bacteria, archaea, fungi, and viruses, their reclassification or renaming of already taxonomically known microbes (Hugon et al., 2016).

### Major bacteria colonizing healthy human gut

Earlier studies aiming to reveal the identity of microbiota in the gut of healthy humans have relied on culturing the microbes from fecal samples. Bacteria constitute most of the microbes colonizing human gut with species belonging to Firmicutes and Proteobacteria phyla being predominant (Eggerth and Gagnon, 1933; Zubrzycki and Spaulding, 1962). To a lesser extent, species belonging to other phyla such as Actinobacteria, Bacteroidetes, and Fusobacteria are also present (Eggerth and Gagnon, 1933; Zubrzycki and Spaulding, 1962). In later studies, several bacterial species, mostly anaerobic, have been identified in fecal samples obtained from 20 healthy individuals using improved cultural techniques (Moore and Holdeman, 1974; Holdeman et al., 1976). At least 400 bacterial species were suggested to colonize human gut and a total of 113 species have been fully recognized in the tested samples (Moore and Holdeman, 1974; Holdeman et al., 1976); major microbes identified being summarized in Tables 1–**5**.

**Table 1:** Firmicutes phylum (class, order, family) and major species constituting the microbiota in healthy human gut.

**Class**	**Order**	**Family**	**Species**	**References**
Clostridia	Clostrdiales	Peptostreptococcaceae	*Peptostreptococcus productus I*	Holdeman et al., 1976
			*Peptostreptococcus productus II*	Moore and Holdeman, 1974; Holdeman et al., 1976
			*Anaerococcus senegalensis*	Lagier et al., 2012
		Ruminococcaceae	*Ruminococcus AJ*	Moore and Holdeman, 1974; Holdeman et al., 1976
			*Ruminococcus albus*	Moore and Holdeman, 1974; Holdeman et al., 1976
			*Ruminococcus obeum*	Holdeman et al., 1976
			(*Blautia obeum*)	Lawson and Finegold, 2015
			*Ruminococcus torques*	Moore and Holdeman, 1974; Holdeman et al., 1976
			*Ruminococcus bromii*	Moore and Holdeman, 1974; Holdeman et al., 1976
			*Ruminococcus massiliensis*	Lagier et al., 2012
		Eubacteriaceae	*Eeubaterium limosum*	Moore and Holdeman, 1974; Holdeman et al., 1976
			*Eubacterium aerofaciens*	Moore and Holdeman, 1974; Holdeman et al., 1976
			(Collinsella aerofaciens)	Kageyama et al., 1999
			*Eubacterium aerofaciens II*	Moore and Holdeman, 1974; Holdeman et al., 1976
			*Eubacterium aerofaciens III*	Moore and Holdeman, 1974; Holdeman et al., 1976
			*Eubacterium siraeum*	Moore and Holdeman, 1974; Holdeman et al., 1976
			*Eubacterium rectile I*	Moore and Holdeman, 1974
			*Eubacterium rectale II*	Moore and Holdeman, 1974; Holdeman et al., 1976
			*Eubacterium rectale III-H*	Moore and Holdeman, 1974; Holdeman et al., 1976
			*Eubacterium rectale IV*	Moore and Holdeman, 1974; Holdeman et al., 1976
			*Eubacterium rectale III-F*	Moore and Holdeman, 1974; Holdeman et al., 1976
			*Eubacterium eligens*	Holdeman et al., 1976
			*Eubacterium biforme*	Moore and Holdeman, 1974; Holdeman et al., 1976
			*Eubacterium formicigenerans*	Holdeman et al., 1976
			*Eubacterium ballii*	Holdeman et al., 1976
			*Eubacterium ventriosum I*	Moore and Holdeman, 1974; Holdeman et al., 1976
			*Eubacterium formicigenerans*	Moore and Holdeman, 1974
			(*Dorea formicigenerans*)	Taras et al., 2002
		Clostridiaceae	*Clostridium leptum*	Holdeman et al., 1976
			*Clostridium ramosum I*	Holdeman et al., 1976
			*Clostridium orbisindens*	Hayashi et al., 2002
			*Clostridium senegalense*	Lagier et al., 2012
			*Faecalibacterium prausnitzii*	Duncan et al., 2002
			*Dorea longicatena*	Qin et al., 2010
		Lachnospiraceae	*Coprococcus eutactus*	Moore and Holdeman, 1974; Holdeman et al., 1976
			*Coprococcus comes*	Moore and Holdeman, 1974
			*Coprococcus catus*	Moore and Holdeman, 1974
		Christensenellaceae	*Christensenella minuta*	Morotomi et al., 2012
			*Christensenella timonensis*	Ndongo et al., 2016a
			*Christensenella massiliensis*	Ndongo et al., 2016b
Bacilli	Lactobacillales	Lactobacillaceae	*Lactobacillus acidophilus*	Rajilic-Stojanovic and de Vos, 2014
			*Lactobacillus leichmannii*	Holdeman et al., 1976
			*Lactobacillus salivarius*	Rajilic-Stojanovic and de Vos, 2014
			*Lactobacillus bulgaricus*	Rajilic-Stojanovic and de Vos, 2014
			*Lactobacillus casei*	Moore and Holdeman, 1974
			*Lactobacillus rhamnosus*	Rajilic-Stojanovic and de Vos, 2014
			*Lactobacillus plantarum*	Rajilic-Stojanovic and de Vos, 2014
			*Lactobacillus fermentum*	Moore and Holdeman, 1974
		Streptococcacaea	*Streptococcus dysgalactiae*	Lagier et al., 2012
			*Streptococcus agalactiae*	Lagier et al., 2012
			*Streptococcus gordonii*	Lagier et al., 2012
	Bacillales	Bacillaceae	*Bacillus massiliosenegalensis*	Lagier et al., 2012
			*Bacillus timonensis*	Lagier et al., 2012
			*Oceanobacillus massiliensis*	Lagier et al., 2012
		Palanococcaceae	*Kurthia massiliensis*	Lagier et al., 2012
			*Kurthia senegalensis*	Lagier et al., 2012
			*Kurthia timonensis*	Lagier et al., 2012
		Paenibacillaceae	*Paenibacillus senegalensis*	Lagier et al., 2012
Erysipelotrichai	Erysipelotrichales	Erysipelotrichaceae	*Dielma fastidiosa*	Lagier et al., 2012; Ramasamy et al., 2013

Until recently, culture-based studies aimed to identify bacterial species in human gut were considered to be limited in their ability to fully evaluate bacterial diversity in human gut. This was based on the fact that such techniques cannot recover all species residing in the human gut (Gerritsen et al., 2011). Lau and colleagues showed that culture-enriched techniques associated with molecular profiling are alternative effective techniques to study microbial diversity in human gut (Lau et al., 2016). Fecal samples from two healthy donors have been cultured aerobically and anaerobically in 33 different culture media creating 66 different culture conditions. Cultured samples were then analyzed using 16S rRNA gene sequencing. In the previous study (Lau et al., 2016), 95% of the bacteria residing human gut has been recovered with 79 new *Lachnospiraceae* isolates being identified. Using a similar approach involving culturing fecal samples obtained from two healthy African donors under 212 different culture conditions, 340 species belonging to seven phyla, and 117 genera have been identified in addition to 24 “novel” microbes (Lagier et al., 2012). Advances in culture conditions have proven to be effective techniques, with currently over than 1,000 microbes reported to be effectively cultured (Rajilic-Stojanovic and de Vos, 2014). These microbes cluster in four main phyla with 450 species in Firmicutes, 214 in Proteobacteria, 164 in Actinobacteria, and 99 in Bacteroidetes (Rajilic-Stojanovic and de Vos, 2014). Collectively, these culture-based studies indicated that human gut is a complex ecological system colonized by thousands of microbes which are quite variable and diverse in-term of their quantity and identity.

Recent studies involving state-of-the-art techniques such as 16S rDNA gene, metagenomic sequencing, and molecular fingerprinting have rapidly increased our knowledge about the identity of the microbes residing in our gut (Hayashi et al., 2002; Knapp et al., 2010; Qin et al., 2010). These studies not only confirmed the identity of the microbes established by cultural techniques, but have also confirmed the incidence on “novel” microbes never detected by culture techniques. Authors characterized several “novel” microbes belonging to *Clostridium* genus in fecal samples obtained from three healthy Japanese individuals by using 16S rDNA libraries and culture-based techniques (Hayashi et al., 2002). In 2004, *Akkermansia muciniphila* a novel mucin-degrading bacterium was also identified from a healthy human volunteer using 16S rRNA-dependent approaches (Derrien et al., 2004). *A. muciniphila* is a Gram-negative bacterium that resides in the mucus layer and constitutes 3–5% of gut microbiota and is thus considered to be the most abundant mucus-degrading bacteria in healthy humans (Schneeberger et al., 2015). Interestingly, the abundance of *A. muciniphila* was found to decrease in obese and diabetic animals following high-fat diet feeding as well as in obese and diabetic human subjects (Schneeberger et al., 2015). In larger scale studies (Qin et al., 2010; Human Microbiome Project Consortium, 2012), the diversity of the microbes in human gut was examined using metagenomic analysis. Species belonging to Firmicutes, Actinobacteria, Bacteroides, and Proteobacteriaphyla were reported (Tables 1–**4**). However, other species belonging to Tenercutes, Spirochaetes, Cyanobacteria, and Verrucomicrobia phyla were reported to be present but at lesser densities (Human Microbiome Project Consortium, 2012).

#### Firmicutes phylum

Firmicutes phylum (Table 1) represents the most diverse and the largest group of microbes making up 60–80% of the total microbes colonizing the GIT of healthy adults. Firmicutes include Gram-positive species with few exceptions such as *Faecalibacterium prausnitzii* (Duncan et al., 2002) previously known as *Fusobacterium prausnitzii* in the phylum Fusobacteria, and *Christensenella minuta* (Morotomi et al., 2012), *C. massiliensis* (Ndongo et al., 2016b), and *C. timonensis* (Ndongo et al., 2016a). Firmicutes phylum is classified into two main classes, Clostridia which includes the genera belonging to *Christensenellaceae, Clostridiaceae, Eubacteriaceae, Lachnospiraceae, Peptococcaceae, Peptostreptococcaceae, Ruminococcaceae*, and *Veillonellaceae* families and Bacilli which are divided into two main orders; Bacillales which includes the genera belonging to *Paenibacillaceae, Planococcaceae, Bacillaceae*, and *Staphylococcaceae*, and Lactobacillales which includes genera belonging to *Aerococcaceae, Carnobacteriaceae, Lactobacillaceae, Leuconstocaceae, Lactococccaceae*, and S*treptococcaceae*. A third class called Erysipelotrichales (e.g., *Dielma fastidiosa*) has been reported to present in human gut (Ramasamy et al., 2013).

#### Proteobacteria phylum

Proteobacteria phylum (Table 2) comprises highly diverse Gram-negative microbes colonizing the GIT of healthy adults. It contains five major classes, α, β, γ, δ, and ε Proteobacteria with γ-class dominating other classes (Shin et al., 2015). Members of the phylum Proteobacteria, mostly belonging to γ-proteobacteria, have a low abundance in the gut of healthy humans (Shin et al., 2015). The γ-proteobacteria constitute the most diverse group of bacteria in the phylum Proteobacteria with six orders and nine families included. Several members in *Aeromonadaceae* and *Vibreonaceae* have been considered as pathogens detected in human intestine (Janda and Abbott, 1998, 2010; Hou et al., 2011). The γ-proteobacteria contain several genera which belong to *Enterobacteriaceae, Pasteurellaceae Succinivibrionaceae, Pseudomonadaceae*, and *Moraxellaceae* families. Many species of *Enterobacteriaceae* are a normal part of the gut flora found in the intestines of humans with their intensity increasing with age (Hopkins et al., 2001). *Escherichia coli* is one of the most important species colonizing the healthy human gut. The healthy human gut contains non-harmful strains of *E. coli*. Several other strains of *E. coli* have been associated with the outbreaks of diarrhea in children (Fagundes Neto et al., 1979; Bratoeva et al., 1994).

**Table 2:** Proteobacteria phylum (class, order, family) and major species constituting the microbiota in healthy human gut.

**Class**	**Order**	**Family**	**Species**	**References**
α-Proteobacteria	Rhizobiales	Hyphomicrobiaceae	*Gemmiger formicilis*	Holdeman et al., 1976
		Methylobacteriaceae	*Microvirga massiliensis*	Caputo et al., 2016
			*Methylobacterium adhaesivum*	Kaakoush et al., 2012
			*Methylobacterium hispanicum*	Kaakoush et al., 2012
			*Methylobacterium radiotolerans*	Lagier et al., 2012
β-Proteobacteria	Burkholderiales	Alcaligenaceae	*Alcaligenes faecalis*	
		Oxalobacteriaceae	*Herbaspirillum massiliensis*	Lagier et al., 2012
		Sutterellaceae	*Parasutterella excrementihominis*	Nagai et al., 2009
			*Sutterella wadsworthensis*	Wexler et al., 1996
			*Parasutterella secunda*	Morotomi et al., 2011
		Neisseriaceae	*Neisseria flavascens*	Lagier et al., 2012
			*Neisseria subflava*	Wang et al., 2005
			*Neisseria preflava*	Lagier et al., 2012
			*Neisseria mucosa*	Lagier et al., 2012
			*Neisseria cinera*	Lagier et al., 2012
γ-Proteobacteria	Aeromondales	Succinivibrionaceae	*Succinatimonas hippie*	Morotomi et al., 2010
			*Anaerobiospirillum succiniciproducens*	Morotomi et al., 2010
	Enterobacteriales	Enterobacteriaceae	*Escherichia coli*	Moore and Holdeman, 1974
			*Escherichia fergusonii*	Lagier et al., 2012
			*Enterobacter massiliensis*	Lagier et al., 2012
			*Enterobacter cloaceae*	Lagier et al., 2012
			*Shigella sonnei*	Hooda et al., 2012
	Pasteurellales	Pasteurellaceae	*Haemophilus parainfluenzae*	Lagier et al., 2012
	Pseudomonadales	Moraxellaceae	*Morexella osloensis*	Lagier et al., 2012
			*Actinobacter radioresistens*	Lagier et al., 2012
			*Actinobacter calcoaceticus*	Lagier et al., 2012
			*Actinobacter septicus*	Lagier et al., 2012
		Pseudomonadaceae	*Pseudomonas aeruginosa*	Lagier et al., 2012
			*Pseudomonas oleovorans*	Lagier et al., 2012
			*Pseudomonas stutzeri*	Lagier et al., 2012
δ-Proteobacteria	Desulfovibrionales	Desulfovibrionaceae	*Desulfovibrio desulfuricans*	Newton et al., 1998
			*Desulfovibrio fairfieldensis*	Loubinoux et al., 2002
			*Desulfovibrio piger*	Loubinoux et al., 2002
			*Bilophla wadsworthia*	Baron et al., 1989
ε-Proteobacteria	Campylobacterales	Campylobacteraceae		
		Halicobacteraceae		

The class δ-proteobacteria includes a branch of predominantly aerobic sulfate-reducing bacteria that belongs to *Desulfovibrionaceae* family (Beaumont et al., 2016). The interest in this class was due to the ability of its species to generate the highly toxic hydrogen sulfide (H_2_S) molecules as part of their metabolic pathways. Overproduction of H_2_S has been associated with pathogenesis of ulcerative colitis and colon cancer (Beaumont et al., 2016).

The class β-Proteobacteria contains species belonging to *Neisseria* genera (Table 2). *Neisseria meningitis* and *Neisseria gonorrhea* are amongst the most studied species classified within this group (Corless et al., 2001; Unemo et al., 2014). Both species are Gram-negative coccoid bacteria, once thought to be restricted to humans and are part of the microflora of the upper respiratory and genito-urinary tracts but have not yet been recognized in the intestine. Despite being considered as part of the normal intestinal flora, *Neisseria subflave* has been linked to meningitis (Baraldes et al., 2000). *Sutterellaceae* species are frequently detected in human gut. They have received much attention in recent year because their density is reported to increase in inflammatory bowel diseases and in children suffering from autism and down-syndrome (De Angelis et al., 2013; Hiippala et al., 2016).

#### Actinobacteria phylum

Actinobacteria phylum (Table 3) constituting healthy human gut microbiota includes diverse Gram-positive species that comprise three orders and 19 families. The *Bifidobacterium* species are one of the major genera of bacteria that are frequently detected exclusively in human gut and have been isolated from infant feces since 1900 (Rajilic-Stojanovic et al., 2007). Some *Bifidobacterium* species are critical to the health of the gut and are now considered as essential constituents of the probiotics used in the treatment of inflammatory bowel disease with no obvious side effects (Ghouri et al., 2014). In contrast to *Bifidobacteriales* species, the *Actinomycetales* species, despite being a highly diverse group of bacteria, have a relatively low abundance in healthy human gut: (10^2^–10^3^) cells/g of fecal sample (Hoyles et al., 2012). Amongst most detected genera are *Corynebacterium* and *Propionibacterium* (Eggerth, 1935; Moore and Holdeman, 1974; Holdeman et al., 1976). Species in *Propionibacterium* are capable of producing propionic acid from lactic acid and also vitamin B12 (Kiatpapan and Murooka, 2002), making them an ideal species to be included in probiotics (Kiatpapan and Murooka, 2002).

**Table 3:** Actinobacteria phylum (class, order, family) and major species constituting the microbiota in healthy human gut.

**Class**	**Order**	**Family**	**Species**	**References**
Actinobacteria	Bifidobacteriales	Bifidobacteriaceae	*Bifidobacterium adolescentis*	Holdeman et al., 1976; Ramirez-Farias et al., 2009; Duranti et al., 2016
			*Bifidobacterium infantis*	Moore and Holdeman, 1974; Holdeman et al., 1976; Hayashi et al., 2002
			*Bifidobacterium longum*	Moore and Holdeman, 1974; Holdeman et al., 1976
			*Bifidobacterium breve*	Moore and Holdeman, 1974
			Bifidobacterium bifidum	Hayashi et al., 2002
	Actinomycetales	Actinomycettaceae	Arcanobacterium haemolyticum	Lagier et al., 2012
			Actinomyces odontolyticus	Lagier et al., 2012
		Dermabacteraceae	Dermabacter hominis	Lagier et al., 2012
		Corynebacteriacaea	*Senegalemassilia anaerobia*	Lagier et al., 2012
			*Corynebacterium appendicis*	Lagier et al., 2012
			*Corynebacterium glaucum*	Lagier et al., 2012
			*Corynebacterium aurimucosum*	Lagier et al., 2012
			*Corynebacterium freneyi*	Lagier et al., 2012
			*Corynebacterium glucuronolyticum*	Lagier et al., 2012
			*Corynebacterium minutissimum*	Lagier et al., 2012
			*Corynebacterium propinquum*	
			*Corynebacterium mucifaciens*	Lagier et al., 2012
			*Corynebacterium tuberculostearicum*	Lagier et al., 2012
			*Corynebacterium coyleae*	Lagier et al., 2012
		Micrococcacaea	*Rothia aeria*	Lagier et al., 2012
			*Rothia dentocariosa*	Lagier et al., 2012
			*Rothia mucilaginosa*	Lagier et al., 2012
			*Micrococcus luteus*	Lagier et al., 2012
			*Kocuria halotolerans*	Lagier et al., 2012
			*Kocuria kristinae*	Lagier et al., 2012
			*Kocuria marina*	Lagier et al., 2012
			*Kocuria palustris*	Lagier et al., 2012
			*Kocuria rhizophila*	Lagier et al., 2012
			*Dermacoccus nishinomiyanensis*	Lagier et al., 2012
			*Arthrobacter castelli*	Lagier et al., 2012
			*Arthrobacter oxydans*	Lagier et al., 2012
		Microbacteriaceae	*Microbacterium oleivorans*	Lagier et al., 2012
			*Microbacterium paraoxydans*	Lagier et al., 2012
			*Microbacterium phylosphaerae*	Lagier et al., 2012
			*Microbacterium schleigeri*	Lagier et al., 2012
			*Microbacterium folliorum*	Lagier et al., 2012
			*Microbacterium gubbeenense*	Lagier et al., 2012
			*Agrococcus jenensis*	Lagier et al., 2012
		Propionibacteriaceae	*Propionibacterium acnes*	Moore and Holdeman, 1974; Holdeman et al., 1976
			*Propionibacterium avidum*	Eggerth, 1935
		Nocardioidaceae	*Aeromicrobium massiliense*	Lagier et al., 2012
			*Rhodococcus equi*	Lagier et al., 2012
			*Rhodococcus rhodocrous*	Lagier et al., 2012
		Gordoniaceae	*Gordonia rubripertincta*	Lagier et al., 2012
		Dietziaceae	*Dietzia natrolonimnaea*	Lagier et al., 2012
			*Dietzia cinnamea*	Lagier et al., 2012
			*Dietzia maris*	Lagier et al., 2012
		Brevibacteriaceae	*Brevibacterium senegalense*	Lagier et al., 2012
			*Brevibacterium linens*	Lagier et al., 2012
			*Brevibacterium epidermidis*	Lagier et al., 2012
			*Brevibacterium halotolerans*	Lagier et al., 2012
			*Brevibacterium iodinosis*	Lagier et al., 2012
			*Brevibacterium casei*	Lagier et al., 2012
			*Brevibacterium ravenspurgense*	Lagier et al., 2012
		Cellulomonadaceae	*Cellulomonas massiliensis*	Lagier et al., 2012
			*Cellulomonas composti*	Lagier et al., 2012
			*Cellulomonas denverensis*	Lagier et al., 2012
			*Cellulomonas parahominis*	Lagier et al., 2012
			*Cellulosimicrobium cellulans*	Lagier et al., 2012
		Sanguibacteraceae	*Timonella senegalensis*	Lagier et al., 2012
		Streptomycetaceae	*Streptomyces missionensis*	Lagier et al., 2012
		Promicromonosporaceae	*Promicromonospora flava*	Lagier et al., 2012
		Micromonosporaceae	*Micromonospora aurantiaca*	Lagier et al., 2012
		Intrasporangiaceae	*Kytococcus schroeteri*	Lagier et al., 2012
	Coriobacteriales	Coriobacteriacaea	*Colinsella aerofaciens*	Lagier et al., 2012
			*Propionibacterium acnes*	Moore and Holdeman, 1974; Lagier et al., 2012
			*Propionibacterium avidum*	Eggerth, 1935
			*Propionibacterium granulosum*	Finegold et al., 1974; Lagier et al., 2012
		Mycobacteriaceae	*Mycobacterium abcsessus*	Lagier et al., 2012
			*Mycobacterium fortuitum*	Lagier et al., 2012

#### Bacteroidetes phylum

Bacteroidetes phylum (Table 4) includes several large classes of Gram-negative, non-spore forming, anaerobic or aerobic, and rod-shaped bacteria that are widely distributed in the environment and in the human guts (Belizário and Napolitano, 2015). By far, species in the class Bacteroidia have been most extensively studied due to their relevance to human metabolic processes. These species play a vital role in metabolizing complex molecules such as proteins and polysaccharides such as cellulose, pectin and xylans into simpler molecules used as source of energy (Wexler, 2007; Xu et al., 2007; Sakamoto and Ohkuma, 2012). Bacteroidia colonizing human gut include species clustered in *Bacteroidaceae, Porphyromonadaceae, Prevotellaceae*, and *Rikenellaceae* families. They are symbiotic and diverse bacteria making up a substantial portion of the normal flora residing in lower GIT (10^10^–10^11^ cells per gram of human feces; Holdeman et al., 1976). Other bacterial species belonging to classes sphingobacteria and flavobacteria have occasionally been detected in healthy human gut (Lagier et al., 2012), however their significance in the gut has yet to be demonstrated.

**Table 4:** Bacteroidetes phylum (class, order, family) and major species constituting the microbiota in healthy human gut.

**Class**	**Order**	**Family**	**Species**	**References**
Bacteroidia	Bacteroidales	Bacteroidaceaae	*Bacteroides thetiotaomicron*	Moore and Holdeman, 1974; Holdeman et al., 1976; Xu et al., 2003
			*Bacteroides fragilis*	Moore and Holdeman, 1974; Holdeman et al., 1976
			*Bacteroides clostridiiformis*	Moore and Holdeman, 1974; Hayashi et al., 2002
			*Bacteroides vulgatus*	Moore and Holdeman, 1974; Holdeman et al., 1976
			*Bacteroides distasonis*	Moore and Holdeman, 1974; Holdeman et al., 1976
			*(parabacteroides distasonis)*	Sakamoto and Benno, 2006
			*Bacteroides capillosus*	Holdeman et al., 1976
			*Bacteroides eggertbin*	Holdeman et al., 1976
			*Bacteroides uniformis*	Hayashi et al., 2002
			*Bacteroides stercoris*	Hayashi et al., 2002
			*Bacteroides eggerthii*	Hayashi et al., 2002
			*Bacteroides goldsteinii*	Moore and Holdeman, 1974
			*(Parabacteroides goldsteinii)*	Sakamoto and Benno, 2006
			*Bacteroides merdae*	Moore and Holdeman, 1974
			*(parabacteroides merdae)*	Sakamoto and Benno, 2006
			*Bacteroides intestinalis*	Qin et al., 2010
			*Parabacteroides distastonis*	Sakamoto and Benno, 2006
			*Parabacteroides johnsonii*	Lagier et al., 2012
			*Parabacteroides merdae*	Sakamoto and Benno, 2006
			*Parabacteroides goldsteinii*	Sakamoto and Benno, 2006
		Porphyromonadacaea	*Gabonibacter massiliensis*	Mourembou et al., 2016
			*Porphyromonas somerae*	Lagier et al., 2012
		Prevotellaceae	*Prevotella copri*	Hayashi et al., 2007
			*Prevotella stercorea*	Hayashi et al., 2007
			*Prevotella oris*	Hasegawa et al., 1997
			*Prevotella bivia*	Lagier et al., 2012
			*Prevotella melalingenica*	Lagier et al., 2012
			*Prevotella nigrescens*	Lagier et al., 2012
			*Prevotella veroralis*	Lagier et al., 2012
			*Prevotella amnii*	Knapp et al., 2010
		Rikenellaceae	*Alistipes senegalensis*	Lagier et al., 2012
			*Alistipes timonensis*	Lagier et al., 2012
			*Alistipes shahii*	Song et al., 2006; Lagier et al., 2012
			*Alistipes obesiensis*	Lagier et al., 2012
			*Alistipes onderdonkii*	Song et al., 2006
			*Alistipes pytredinis*	Hayashi et al., 2002
Sphingobacteria	Sphingobacteriales	Sphingobactereaceae	*Sphingobacterium multivorum*	Lagier et al., 2012
Flavobacteria	Flavobacterialis	Flavobacteriaceae	*Flavobacterium lindanitolerans*	Lagier et al., 2012

#### Fusobacteria phylum

Fusobactera phylum (Table 5) includes Gram-negative, non-sporeforming, anaerobic bacilli frequently detected in human gut (Walter et al., 2002). This phylum includes species belonging to *Fusobacteriaceae* and *Leptotrichiaceae* families. Species within *Fusobacteriaceae* were considered to be limited to the oral cavity until 1966 when it was shown that they could be detected in fecal samples (Van Houte and Gibbons, 1966). *Fusobacteriaceae* species appear to be directly related to the health of the gut. Their density increases in inflammatory diseases such as appendicitis (Swidsinski et al., 2012), ulcerative colitis (Rajilic-Stojanovic et al., 2013) and colon cancer (Kostic et al., 2012). *Leptotrichiaceae* species appears to be detected mainly in human elderly gut (Hayashi et al., 2005) and female reproductive system (Thilesen et al., 2007).

**Table 5:** Fusobacteria phylum (class, order, family) and major species constituting the microbiota in healthy human gut.

**Class**	**Order**	**Family**	**Species**	**References**
Fusobacteria	Fusobacteriales	Fusobacteriaceae	*Fusobacterium prausnitzii*	Moore and Holdeman, 1974; Holdeman et al., 1976; Miquel et al., 2013
			*Fusobacterium russii*	Moore and Holdeman, 1974; Holdeman et al., 1976
			*Fusobacterium varium*	Holdeman et al., 1976; Walter et al., 2002
			*Fusobacterium gonidiaformans*	Walter et al., 2002
			*Fusobacterium naviforme*	Walter et al., 2002
			*Fusobacterium mortiferum*	Holdeman et al., 1976
			*Fusobaccterium nucleatum*	Holdeman et al., 1976
			*Fusobaccterium peridonticum*	Roberfroid et al., 2010
		Leptotrichiaceae	*Leptotrichia buccalis*	Vaahtovuo et al., 2005
			*Leptotrichia amnionii*	Rajilic-Stojanovic and de Vos, 2014

### Archaea, viruses, and fungi colonizing healthy human gut

Healthy human gut microbiota includes archaea, fungi, and viruses as consistent residents (Parfrey et al., 2011; Lloyd-Price et al., 2016; Rehman et al., 2016), even though in some cases at low densities. A small number of archaeal genera have been identified in the healthy human microbiota, primarily in the gut. Historically, archaea were classified as bacteria with the name (archaebacteria) before being reclassified in a specific domain (Woese et al., 1990; Pace, 2006), since they have characteristic features unique enough to separate them from bacteria and Eukaryota domains (Cavicchioli, 2011). In total, eight archaeal species have been associated with the human GIT (Rajilic-Stojanovic and de Vos, 2014). Species of the Methanobrevibacter genus, namely; *Methanobervibacter smithii, M. ruminantium, M. stadtmaniae*, and *M. luminyensis*, are the most prevalent (Rajilic-Stojanovic and de Vos, 2014; Horz, 2015) in the healthy gut. *M. smithii* in particular, has been found to be well-adapted to inhabit the human gut. These species are implicated in optimizing the digestion process of dietary polysaccharides in association with other microbes (Samuel et al., 2007) and they are capable of producing methane (methanogenes) from CO_2_ and H_2_. Their abundance in infants is relatively low (10^2^–10^6^ cell/g of fecal sample) and increases during adulthood reaching up to 10^10^ cells/g fecal samples. Such changes in densities explain the absence of methane gas, as detected by a breath test, during infancy in contrast to adulthood (Gaci et al., 2014).

Archaeal science appears to be in its early stages. Unlike bacteria, archaeal species are largely ignored as a topic in microbiology, possibly due to the lack of appropriate genomic tools to reveal their existence and their diversity (Horz, 2015). Accordingly, it is quite likely that the archaeal domain represents a diverse community with several yet unknown taxa, waiting to be identified. Although, some archaeal species (methanogens) appears to be associated with gut inflammatory diseases, including constipation, the question arises as to whether it is necessary to identify all taxa. Even if they are rare and/or present only in very low abundance, their precise role in disease development remains unknown (Gaci et al., 2014; Horz, 2015).

Many viral species have been reported to colonize the healthy human gut system forming a symbiotic relationship with their bacterial (bacteriophages) and human hosts. These viruses constitute the so called “human gut virome” (Zou et al., 2016). Each individual holds a unique viral profile, highlighting the high interpersonal variability of the healthy human gut virome (Minot et al., 2011; Scarpellini et al., 2015). The human virome includes viruses from seven families, namely Herpesviridae, Polyomaviridae, Papillomaviridae, Adenoviridae, Anelloviridae, Parvoviridae, and Circoviridae. Not all families have been detected in healthy human gut (Wylie et al., 2014). In a large scale metagenomic study involving 102 healthy individuals, roseolovirus (Herpesviridae), alphapapillomavirus and gammapapillomavirus (Papillomaviridae), mastadenovirus (Adenoviridae), polyomavirus (Polyomaviridae), gyrovirus (Circoviridae), and some other unclassified viruses were generally detected in fecal samples (Wylie et al., 2014).

A better understanding of human virome composition and dynamics should confirm that it is an important factor contributing to human health (Scarpellini et al., 2015). Indeed, several recent studies have implicated gut virome in regulating/stabilizing their host bacterial species and subsequently maintaining microbial diversity in the gastrointestinal tract (Minot et al., 2011; Abeles and Pride, 2014; Scarpellini et al., 2015).

In addition, several fungal species have been reported to colonize healthy human gut, even if at low amounts (Ott et al., 2008; Sokol et al., 2016). The most prevalent fungal species detected in the healthy human gut are clustered into three phyla; Ascomycota, Basidiomycota, and Zygomycota. *Candida albicans* and *Candida rugosa* have routinely been detected in human gut (Ott et al., 2008; Sokol et al., 2016). Most *Candida* species under normal conditions form symbiotic/commensal relationships with the host. However, when host environmental conditions favor the outgrowth of *C. albicans*, excessive colonization can lead to infection and invasion of host tissues (Ott et al., 2008; Sokol et al., 2016).

## Diversity along human gut

The human gut is colonized by higher numbers of bacteria and more diverse species compared to other parts of the body (Quigley, 2013). The microbial composition of the gut flora varies along the gut (Table 6), with stomach and small intestine having relatively lower microbial diversity (Guarner and Malagelada, 2003; O'Hara and Shanahan, 2006). In contrast, the colon is densely colonized by microbes reaching up to 10^12^ cells per gram of intestinal content (O'Hara and Shanahan, 2006). There are between 300 and 1,000 different bacterial species, but the vast majority of bacteria (99%) come from 50 to 60 species (Guarner and Malagelada, 2003; Rajilic-Stojanovic et al., 2007). Because of their abundance in the intestine, bacteria represents ~60% of fecal dry mass (Stephen and Cummings, 1980). Fungi, archaea, and viruses are also present in the gut flora, but less is known about their activities (Lozupone et al., 2012).

**Table 6:** Diversity of microbes along the human gut.

	**Stomach**	**Duodenum**	**Jejunum**	**Proximal ilium**	**Distal ilium**	**Colon**	**References**
pH	1.4–5	4.5–6.1	4.7–6.5	6.3–7.4	6.8–7.9	5.3–6.7	Evans et al., 1988
Food passage time (h)	2–6	3–5				10–20	
Microbial density /g sample	−10^2^	10^2^	10^2^	10^3^	10^8^	10^12^	O'Hara and Shanahan, 2006
Major genera	*Helicobacter pylori*	*Streptococcus Lactobacillus*	*Streptococcus Lactobacillus*	*Streptococcus Lactobacillus*	*Streptococci*	*Bacteroides*	Davis, 1996; Gaci et al., 2014; Scarpellini et al., 2015
					*Lactobacilli*	*Bifidobacterium*	
					*Bacteroides bifidobacteria*	*Eubacterium, Peptostreptococcus*	
	*Streptococcus*						
	*Clostridium*						
	*Staphylococcus*					*Virus*	
	*Lactobacillus Peptostreptococcu*					*Fungi*	
						*Archaea*	
Number of phylotypes	NA	NA	22	NA	33	37	Wang et al., 2005

### Stomach flora

Healthy human stomach has no longer been considered as a “sterile organ” since the discovery of *H. pylori* (Marshall and Warren, 1984). It is rather now considered as a harbor for many bacterial species, dominated by five major phyla: Firmicutes, Bacteroidites, Actinobacteria, Fusobacteria, and Proteobacteria. Generally, the healthy human stomach is dominated by Prevotella, Streptococcus, Veillonella, Rothia, and Haemophilus (Bik et al., 2006; Zilberstein et al., 2007). Characterizing healthy stomach microbiota has come from studies involving gastric biopsies and stomach juices collected from individuals routinely performing upper gastrointestinal endoscopy for dyspepsia (Li et al., 2009; Engstrand and Lindberg, 2013). Using a small subunit 16S rDNA clone library approach (Bik et al., 2006), 128 phylotypes have been shown to colonize the stomach, coming from the five major phyla mentioned above. Essentially similar findings have been reported by other researchers despite differences in techniques (Delgado et al., 2013) and the ethnic background of biopsy donors (Li et al., 2009; Delgado et al., 2013; Engstrand and Lindberg, 2013), suggesting that some homogeneity may exist in the composition of stomach microbiota.

The contribution of stomach microbiota to disease pathogenesis has not yet been fully explored, although an alteration in the density of Firmicutes phylum, particularly the Streptococcus and Prevotella genera, has been reported in patients with *H. pylori* infection and stomach cancer (Wroblewski and Peek, 2016). It remains pivotal to know to what extent *H. pylori* infection or stomach cancer affects the composition of stomach microbiota and whether restoring stomach biota, either naturally or pharmacologically, can modulate the outcome of an *H. pylori* infection or cancer progression (Wroblewski and Peek, 2016).

### Intestinal flora

Most of the studies describing gut microbial composition in health adults have usually involved fecal samples, representing the diversity of the microbes present in the large intestine (Finegold et al., 1974; Moore and Holdeman, 1974; Holdeman et al., 1976). The small intestine, however, is more acidic, has higher levels of oxygen, less transit time, and has effective immune-cell-mediated antimicrobial factors compared with the colon (Keshav, 2006). Such physiological variations promote less microbial diversity in small intestine compared with colon. Accordingly, only fast growing facultative anaerobes, which tolerate the bactericidal effects of bile acid and effectively compete for carbohydrate, will dominate this part of the gut (Zoetendal et al., 2012). There are limited studies aimed to assess small intestinal microbial diversity (Hartman et al., 2009; Zoetendal et al., 2012). This is partly due to the technical difficulties in collecting samples for analysis.

Small intestine microbiota has been examined in three different anatomical locations, namely jejunum, ileum, and distal ileum. Despite the fact that each part is characterized by its own microbial composition, it appears that the profile of the jejunum is closely related to that of the stomach; with Bacilli, mainly of the *Streptococcaceae* species, dominating this section (50–70%) (Wang et al., 2005; Zoetendal et al., 2012). Even in elderly, moving along the small intestine toward the colon, the intensity of Bacilli species in the ileum and distal ileum drop remarkably to reach 20 and 5%, respectively (Hayashi et al., 2005; Zoetendal et al., 2012). Instead Clostridia species such as IX, XIVb XIVa, and IV form up to 30% of the microbiome, while Bacteroidetes species occupy a proportion of 49% at these locations (Wang et al., 2005; Zoetendal et al., 2012).

## Human gut microbiota in human health and disease

There is increasing evidence that differences in the structure, function, and diversity of the human gut microbiota are associated with states of human health and disease.

### Establishment of gut microbes

The first 2 years of life mark a dynamic period in which the gut microbiome builds from the initial microbial repository at birth and adjusts until the composition and function are more like that of an adult (Koenig et al., 2011). The type of birthing delivery strongly influences which microbes are present upon the initial establishment of an individual's gut microbiota. Infants born vaginally develop gut microbiota that are more similar to their mothers than those born via cesarean section; this also conferring some functional differences. For example, gut microbiota in infants born vaginally tend to express a lower proportion of antibiotic resistance genes (Bäckhed et al., 2015). The developing gut microbiota is strongly influenced by the infant's diet and life events. Over time, the infant begins harboring microbes capable of digesting complex sugars and starch (Koenig et al., 2011; Bäckhed et al., 2015). As more types of foods are introduced into the infant's diet, the bacterial diversity in the gut increases. For infants who are breastfed, the discontinuation of breastfeeding appears to be the strongest factor driving the change in gut microbiota structure from the less diverse infant microbiome to the more diverse adult phenotype (Koenig et al., 2011; Bäckhed et al., 2015).

Host genetic signature has also been reported to contribute, to a certain extent, to the types of microbes present within an individual's gut. Although, studies generally suggest that genetic effects do not exert a strong global influence on which microbes colonize the gut, monozygotic twin pairs display more similar microbiota than their dizygotic counterparts (Turnbaugh et al., 2009; Smith et al., 2013; Goodrich et al., 2014). Moreover, even if few bacterial taxa exhibit strong heritability, the strongest are associated with clinically meaningful phenotypic differences (Goodrich et al., 2014). Recent evidence suggests that there is cross talk between the gut microbiome and host genetic signature that results from altered gene expression (Richards et al., 2016). In other words, differential gene expression in host cells allow for specific types of bacteria to colonize one individual over another, and the way a cell responds to bacteria (or the community of microbes present) may vary depending on the host's genetic make-up. However, environment and diet appear to be the strongest drivers of microbiota composition, with some of the observed variance attributable to host genetics (Richards et al., 2016).

### Gut microbiota and human health

Despite inter-individual differences in the structure and diversity of the human gut microbiome, the microbial metabolic and functional pathways remain stable among healthy individuals (Human Microbiome Project Consortium, 2012). The human gut microbiome encodes at least 10-fold more genes than the human genome and functional redundancy among some of these genes allow different microbes to create individualized communities that will carry out the same functions to maintain homeostasis and a symbiotic relationship with the human host (Ley et al., 2006; Qin et al., 2010). The functional redundancy is important for maintaining a favorable environment within the gut to ensure survival of the bacteria, while also contributing to human metabolism and health (Parekh et al., 2014). This is significant because the bacteria present in gut microbiome communities help liberate carbohydrates and other nutrients from the diet that could otherwise not be utilized by the human host (Larsbrink et al., 2014). For example, certain species of Bacteriodetes can metabolize xyloglucans, a complex carbohydrate found in dietary fiber that contributes to human's carbohydrate intake. At least one of the taxa capable of this type of metabolism is consistently reported in studies evaluating gut microbiota (Larsbrink et al., 2014). Since products of gut microbe metabolism, like that of xyloglucans, contribute to the symbiotic relationship between host and microbe, there has been increasing interest in how the microbes and their metabolic products contribute to human health and disease.

As mentioned, Firmicutes and Bacteroidetes are the most predominant phyla in the human gut and affect the production of short-chain fatty acids (Rosenbaum et al., 2015). Short-chain fatty acids (SCFA) produced from indigestible carbohydrates by gut microbes are used as energy by various human tissues (Rosenbaum et al., 2015). Some studies have observed that the proportions of bacteria present in these phyla may contribute to metabolic outcomes in the host, in part by altering the amount of short-chain fatty acids produced (Turnbaugh et al., 2006, 2009). Greater density of Bacteroidetes has been associated with increased butyrate and propionate levels that contribute to a healthy body weight by suppressing hunger and helping to maintain glucose homeostasis (Lin et al., 2012). Butyrate produced by the gut microbes also contributes to health by providing energy for colonic epithelial cells and inhibiting inflammation, particularly that induced by lipopolysaccharide (Cani et al., 2007; Belkaid and Hand, 2014).

*A. muciniphila*, is a Gram-negative bacterium of the order Verrucomicrobiales. Despite containing lipopolysaccharides (LPS), a well-known endotoxin, a study by Everard et al. (2013) has demonstrated the lack of direct association between Gram-negative bacteria and gut or metabolic endotoxemia (Everard et al., 2013). On the contrary, oligofructose administration which has been reported to restore *A. muciniphila* levels mitigated endotoxemia caused by HFD (Everard et al., 2013). Other studies also reported the seemingly counterintuitive results that *A. muciniphila* preserves the integrity of the intestinal mucous and intestinal barrier function and counteracts the deleterious effect of HFD on gut permeability despite having week mucin-degrading activities (Cani et al., 2009; Belzer and de Vos, 2012; Everard et al., 2013). One explanation is that this bacterium protects against inflammation through increasing the levels of the anti-inflammatory intestinal endocannabinoids which control gut barrier and gut peptide secretion (Everard et al., 2013). In addition, higher levels of *A. muciniphila* were associated with greater enteroendocrine L-cell activity, hence more secretion of glucagon-like peptides GLP-1 and GLP-2 (Cani et al., 2009). Gut peptides including GLPs were reported to control epithelial barrier proliferation and integrity (Belzer and de Vos, 2012). It was demonstrated that *A. muciniphila* controls GLP-2 secretion through increasing levels of 2-oleoylglycerol (2-OG) and reduces metabolic endotoxemia via increasing 2-arachidonoylglycerol (2-AG) levels (Everard et al., 2013). However, the link between the enhanced production of endocannabinoids and the beneficial effects of *A. muciniphila* needs further investigation. Other studies reported that *A. muciniphila* levels were reduced in diseases involving dysfunctional intestinal barrier such as irritable bowel syndrome (IBS) and chronic granulomatous colitis (Falcone et al., 2016). However, some authors reported contradictory results; for example a Chinese MGWAS study stated that *A. muciniphila* did not improve mucous layer thickness (Tilg and Moschen, 2014), while Ganesh et al. attributed exacerbated inflammation in *Salmonella typhimurium*-infected mice to the mucin-degrading effect of *A. muciniphila* (Ganesh et al., 2013). Such conflicting studies need further clarification.

In general, imbalances in the types of bacteria present in the gut microbiota are thought to contribute to disease in part by altering different metabolic processes and/or pathways in the host.

### Gut microbiota and disease

Differences in the composition of the gut microbiota in humans that relate to disease have been reported for several conditions including, but not limited to, cardiovascular disease (Holmes et al., 2008; Wang et al., 2011), type 2 diabetes (Larsen et al., 2010), and obesity (Turnbaugh et al., 2009). Interest in studying the human gut microbiota related to disease has increased, in part due to the fact that it appears to be highly influenced by interventions that ameliorate symptoms or disease such as medications and diet. For example, individuals that eat red meat exhibit greater levels of the gut metabolite trimethylamine-N-oxide (TMAO) than non-meat eaters. Importantly, TMAO is associated with increased plaque formation in arteries (Koeth et al., 2013; Tang et al., 2013). The fact that products from microbial metabolism play an integral role in many metabolic pathways of the host, suggests that complex disorders with metabolic components may benefit from targeted alterations in the microbiota through dietary or supplement interventions. For this review, we will focus specifically on reviewing the microbiome literature as it relates to metabolic syndrome.

### Overview of metabolic syndrome

Metabolic syndrome is a group of factors that collectively raise the risk for other chronic and acute disease processes. The greater the number of the risk factors an individual has, the higher the risk for other poor health outcomes and disease such as heart attacks, stroke, and type 2 diabetes. Metabolic syndrome risk factors are closely linked with obesity and sedentary lifestyles (Alberti et al., 2009; Kaur, 2014). There are several different reports that include diagnostic criteria for metabolic syndrome that are utilized in different areas of the world (Alberti et al., 2009; Kaur, 2014). Despite some minor difference in criteria, individuals presenting with metabolic syndrome exhibit 2–3 of the following characteristics: central obesity, abnormal serum lipid levels (high triglycerides and/or low high-density lipoprotein), high blood pressure, and elevated blood glucose levels. Some groups contain diagnostic metrics for insulin resistance, whereas others do not since it is difficult to uncouple from obesity (Alberti et al., 2009; Kaur, 2014).

The mechanistic pathways for obesity are complex due to a multiplicity of genetic and lifestyle factors that contribute to weight gain, although insulin is a critical regulator of adipocyte biology (Grundy et al., 2004). Insulin inhibits lipolysis and stimulates glucose transport as well as triglyceride synthesis (Grundy et al., 2004). When lipolysis is stimulated in insulin resistant individuals, large amounts of circulating fatty acids and inflammatory markers are released from expanded adipose tissue mass (Grundy et al., 2004; Afsar and Elsurer, 2014). When these processes occur consistently over time, elevations in weight and blood sugar become apparent and begin to influence other metabolic processes (Grundy et al., 2004; Afsar and Elsurer, 2014). The increase in circulating free fatty acid levels contributes to the development of triglyceride reservoirs in muscle and liver, resulting in decreased glucose uptake in muscle, increased hepatic gluconeogenesis, and increased blood cholesterol and triglyceride levels which may also contribute to rising blood pressure (Afsar and Elsurer, 2014). The resultant pro-inflammatory and pro-thrombotic states increase the risk for cardiovascular disease and type 2 diabetes (Afsar and Elsurer, 2014). Since the aforementioned components of metabolic syndrome overlap to some degree mechanistically, similar overlap in differences in microbiota have been reported based on metabolic syndrome diagnostic criteria (Rial et al., 2016).

### Gut microbiota and obesity

Diagnostic criteria for obesity, particularly central or abdominal obesity, vary by guidelines and populations being assessed. Despite differences in diagnostic cutoffs determining obesity, several research groups have reported differences in gut microbiota related to body weight (Turnbaugh et al., 2009). Some of the first ground breaking studies investigating the gut microbiota in humans sought to evaluate the relationship with obesity. The first of which identified a decrease in diversity of gut microbiota in obese individuals, whereas this diversity was increased with dietary weight loss (Turnbaugh et al., 2009). A meta-analysis found that studies tend to report shifts in different microbes associated with obesity (Walters et al., 2014). This may be related to the functional redundancy in the gut microbiota, since multiple different taxa can contribute to the same metabolic pathway. Walters and colleagues also noted that some of the variability of results reported between studies was due to differences in laboratory protocols and analytic pipelines. For example, in the Turnbaugh and colleagues twin study (Turnbaugh et al., 2009), a *de novo* approach was used which assigns operational taxonomic units (OTUs) without an external reference. In the meta-analysis assessing results of obesity associated microbiome changes using a closed-reference OTU assignment process, where sequences that do not match a reference data set are excluded; there were no significant differences in the microbiota between obese and lean individuals (Turnbaugh et al., 2009). In addition to some of equivocal results observed when different laboratory and data processing pipelines are used, there is the question of the reproducibility of murine studies in humans because there are significant differences in how the different species maintain energy homeostasis as diet and metabolic demands are different (Turnbaugh et al., 2009). There are few studies evaluating processes identified as contributing to obesity in murine models and in human populations, the same applies to most of the components of metabolic syndrome (Rosenbaum et al., 2015). Studies are increasingly evaluating host genetic signatures as well as the microbiota to address these issues. Studies designed in this manner have identified features in the gut microbiome that suggest a strong relationship between certain microbes and host genomic loci that are linked to obesity in human populations (Le Chatelier et al., 2013). Despite this ambiguity, continuing studies of the human gut microbiota aim to refine result of the work that has been done thus far.

### Gut microbiota and dyslipidemia

All diagnostic guidelines for metabolic syndrome include metrics of dyslipidemia, particularly high levels of triglycerides and low levels of high-density lipoprotein (HDL) (Kaur, 2014). All consistently categorize triglycerides as elevated when serum levels are above 150 mg/dL, and there is some variability between guidelines about what constitutes low HDL levels among men and women (Kaur, 2014). Some bacterial taxa from the human gut are present in artherosclerotic plaques, although the community of microbes within the plaque most closely resembles bacterial taxa that predominate in the oral cavity (Koren et al., 2011). Two uncharacterized taxa in the human gut, from the Erysipelotrichaceae and Lachnospiraceae families, were correlated with total cholesterol and LDL levels, but no association was observed related to serum HDL levels (Koren et al., 2011). A recent study evaluating the relationship between the human gut microbiota and blood lipid levels found that the gut microbiota has significant differences in the same metrics that are characteristics of metabolic syndrome, namely obesity, serum triglyceride, and HDL levels (Fu et al., 2015). Unlike Koren's team, Fu and colleagues did not find any variability in the gut microbiota composition to be associated with elevated levels of low-density lipoproteins (LDL) or total cholesterol levels, which are not included as diagnostic criteria for metabolic syndrome (Fu et al., 2015). Fu's team identified 34 bacterial taxa that were associated with obesity, triglyceride and HDL levels in their study group. Further, they determined that the gut microbiota composition accounted for nearly 26% of the variance observed in HDL levels. These data suggest that treatments aimed at altering the gut microbiota to increase levels of HDL cholesterol may have potential to be highly effective (Fu et al., 2015).

### Gut microbiota and blood sugar

Obesity and adiposity are directly related to increased risk for elevated blood glucose levels and type 2 diabetes (Alberti et al., 2009). All diagnostic guidelines contain metrics for impaired fasting glucose or impaired glucose tolerance, with or without diabetes (Kaur, 2014). Some guidelines for metabolic syndrome use glucose metrics interchangeably with insulin resistance metrics (Kaur, 2014). Differences in gut microbiota have been reported in individuals with type 2 diabetes, with elevated levels of Bacteroidetes and Proteobacteria and lower levels of Firmicutes than healthy individuals (Larsen et al., 2010). Interestingly, in Larsen's study, they did not observe the commonly reported decreased level of Bacteroidetes in individuals that were diabetics and also obese. However, others have observed that the composition of the gut microbiota in obese individuals with insulin resistance or elevated glucose levels agree with previous studies reporting lower levels of Bacteroidetes and butyrate producing species (Qin et al., 2012; Vrieze et al., 2012). As discussed previously, obesity can contribute to the development of this phenotype by increasing insulin resistance, resulting in higher levels of glucose remaining in the serum. A small intervention study (*N* = 18, *n* = 9/group) reported an increase in bacterial diversity in the gut when microbiota from lean donors was transplanted into obese recipients; with an associated increase in butyrate producing bacteria and subsequent increase in insulin sensitivity (Vrieze et al., 2012).

*A. muciniphila* levels were found to be inversely associated to obesity and diabetes in several animal and human studies (Tilg and Moschen, 2014). The role of *A. muciniphila* in reducing inflammation could provide protection against the development of type 2 diabetes (Schneeberger et al., 2015). In addition, administration of *A. muciniphila* reduced body weight, adipose tissue inflammation, lipidemia, and hyperglycemia in diabetic and obese animals and increased adipose tissue browning and fatty acid oxidation (Tilg and Moschen, 2014). However, two recent studies reported increased levels of *A. muciniphila* in animals fed with high fat and high carbohydrate diet (Carmody et al., 2015; Hamilton et al., 2015). In human studies, it was found that under certain levels of *A. muciniphila*, human subjects responded less efficiently to caloric restriction diet in terms of reduction of hypergycemia, insulin resistance and inflammatory markers (Hamilton et al., 2015). However, there is no simple relationship between levels of *A. municiphila* and inflammation, and the threshold beyond which a shift occurs between healthy to pathological conditions is still unknown (Hamilton et al., 2015).

Together, these studies suggest that the composition/diversity of gut microbiota may contribute to elevated glucose levels and insulin resistance in individuals with metabolic syndrome.

### Gut microbiota and metabolic syndrome

Many of the studies evaluating the gut microbiota related to the risk factors associated with metabolic syndrome report associations among multiple features of metabolic syndrome (Rial et al., 2016). Given that multiple risks occur concurrently, even in the absence of metabolic syndrome (i.e., having only 2 of the associated risk factors), it is difficult to disentangle the individual contribution to disease state without considering confounding factors. Some of the associations with multiple risk factors suggest that the gut microbiome is intimately involved with observed variations over and above those related to age, sex, and host genetics (Rial et al., 2016). Notably 12 bacterial OTUs have been associated with variability across three traits (BMI, triglycerides, and HDL levels) (Fu et al., 2015). Others have found that lower diversity associated with obesity is also associated with insulin resistance and dyslipidemia (Le Chatelier et al., 2013).

Because of confounding of characteristics associated with obesity, dyslipidemia, and elevated blood glucose levels, some scientists have evaluated the human gut microbiota composition specifically related to metabolic syndrome. Similar to what is observed in obese individuals by Turnbaugh's group (Turnbaugh et al., 2009), a lower level of diversity of gut microbes was present in individuals with metabolic syndrome; and some of the taxa associated with metabolic syndrome are linked to a genetic variant in the apolipoprotein A5 gene (*APOA5*) (Lim et al., 2016). Specifically, Lim and colleagues noted that specific taxa associated with metabolic syndrome were not all correlated with each characteristic of metabolic syndrome in the same way. For example, they found *Lactobacillus* to be correlated with central obesity and fasting blood sugar, but negatively correlated with HDL levels (Lim et al., 2016). This suggests that metabolites from gut metabolism contribute to multiple mechanistic pathways involved in metabolic syndrome and that there may be different combinations of contributing microbes depending on which characteristics of metabolic syndrome are present in an individual (Lim et al., 2016). This suggests that multiple interventions may be effective for treating and preventing metabolic syndrome by targeting mechanistic pathways associated with different characteristics of metabolic syndrome.

## Therapeutic modulation of gut microbiota to restore lipid and glucose homeostasis

### Polyphenols

Polyphenols constitute a large group of heterogeneous secondary metabolites found almost ubiquitously in the plant kingdom. The daily intake of dietary phenols is estimated to be larger than 1 g, which is 10 times higher than vitamin C intake from diet (Scalbert et al., 2005). Their chemical structures are characterized by the presence of polyhydroxyphenyl units and range from simple monomers and oligomers to highly polymeric compounds with molecular weight reaching up to 30,000 Da, such as condensed tannins (Tsao, 2010). Polyphenols can be further classified according to their chemical structures into two subgroups: flavonoids and non-flavonoid polyphenols, including phenolic acids. They predominantly exist in combination with sugars or acylated sugars (glycosides), but may also occur in other conjugated structures (amides, esters, and methyl ethers) or in their free forms (Tsao, 2010).

**Figure 2:**
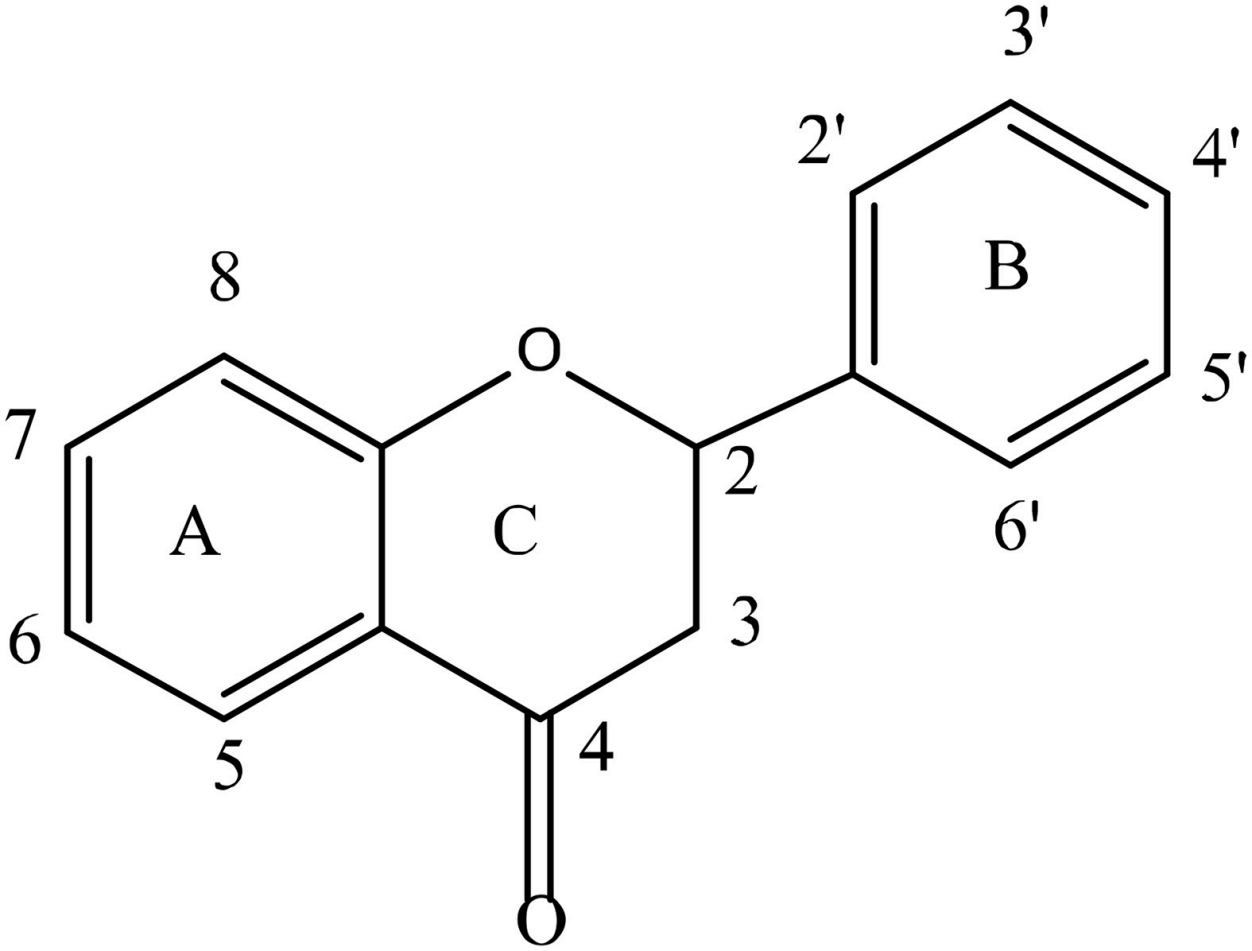
The basic structure of flavonoids.

**Figure 3:**
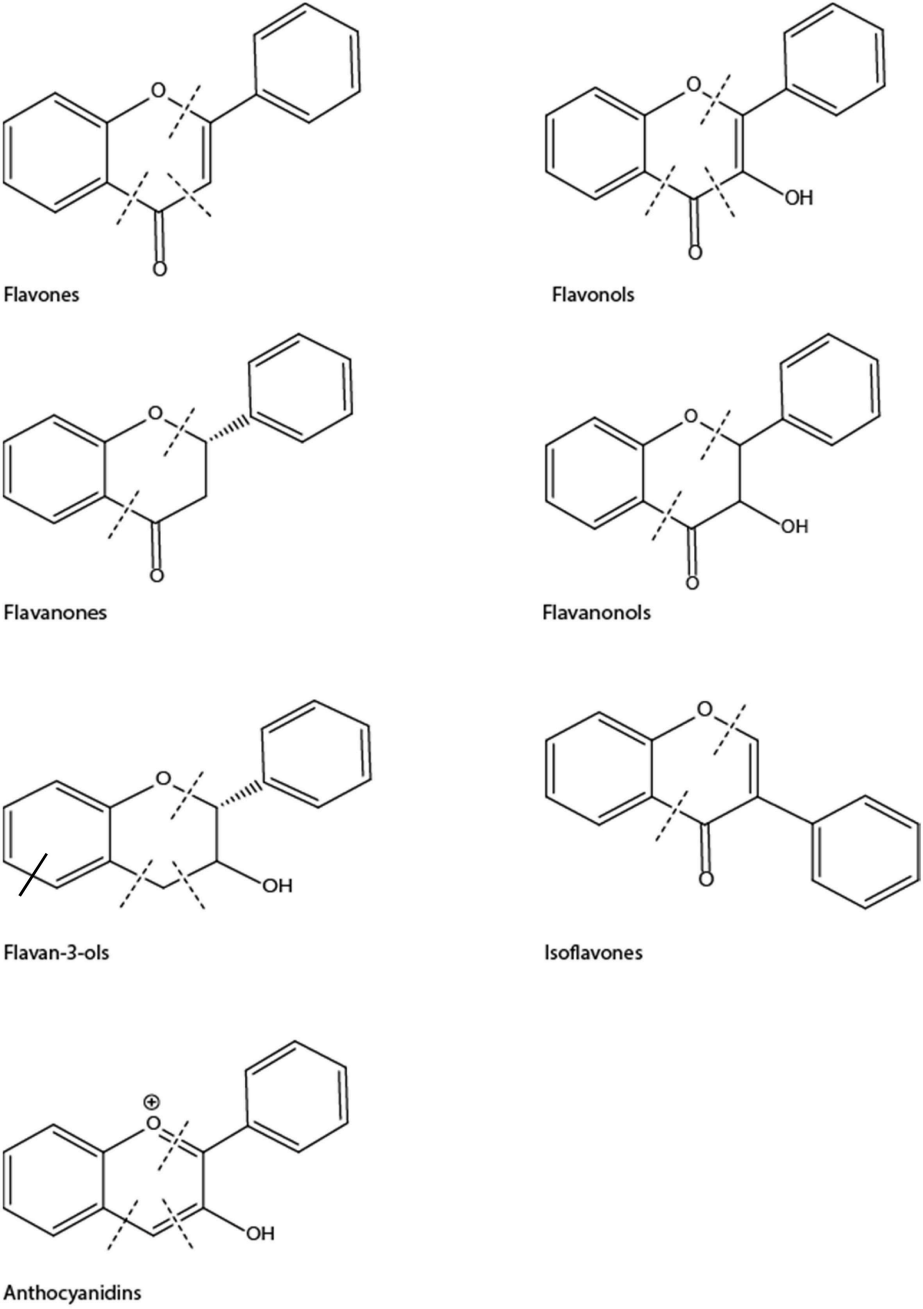
Chemical structures of main classes of flavonoids showing positions of potential C-ring cleavage (——) or A-ring cleavage (——).

Flavonoids form the largest group of polyphenolic compounds with more than 6000 compounds identified and/or isolated from plant sources (Kumar and Pandey, 2013). For a polyphenol to be classified as a flavonoid, its structure has to contain a benzo-pyrane (chromane) moiety included in a C6-C3-C6 structural backbone (Figure 2), in which the two C6 units are benzyl rings (ring A and ring B) and C3 unit is the chromane ring (ring C). Depending on the hydroxylation and substitution patterns, as well as the degree of saturation of the chromane ring, flavonoids can be further divided into several classes such as: flavones, flavonols, flavanones, flavanonol, flavan-3-ols, anthocyanins, and isoflavones (Figure 3). Condensed tannins, also known as proanthocyanidins or polyflavonoid tannins, are polymers of flavan-3-ols or flavan-3,4-diols (Figure 3). In all flavonoid classes, the ring B is attached to the carbon-2 of ring C; with the exception of isoflavonoids in which the attachment between the two rings takes places at carbon-3 of ring C. Interestingly, this particular arrangement has conferred isoflavones the ability to interact with estrogen receptor and to act as weak phytoestrogens (Ozdal et al., 2016).

The nonflavonoid-polyphenols have more heterogeneous structures and broadly comprises hydrolysable tannins (gallotannins and elagitannins), lignans stilbenes, and phenolic acids). Phenolic acids in food are mainly benzoic acid or cinnamic acid derivatives (Lafay and Gil-Izquierdo, 2008). Some phenolic acids such as *p*-coumaric and hydroxyl-cinnamic acids are considered “functional polyphenols” despite their monophenolic structures because they share many properties with polyphenols (Pereira et al., 2009).

Polyphenols are produced in plants to serve important roles such as protection against different environmental stressors and pathological aggressions, thus acting as primary defense mechanisms (phytoalexins). For this reason, polyphenols are endowed with excellent antioxidant, antifungal, antibacterial, and photo-protective properties (Li et al., 2014).

Over the last few decades, research on polyphenols has expanded exponentially and many other biological activities and health benefits have been attributed to polyphenols. These include, but are not limited to, anti-inflammatory, anticancer, and protective activities against inflammatory and oxidative stress-induced disorders such as aging, rheumatoid arthritis, diabetes mellitus, cardiovascular and neurodegenerative diseases (Li et al., 2014). As data accumulated from research studying the mode of actions of polyphenols, it soon became clear that their health benefits go beyond antioxidant potential (Scalbert et al., 2005).

Scientific interest in polyphenols has rapidly grown in the late twentieth century following many epidemiological studies linking the consumption of polyphenol-rich food such as fruits and vegetables to lower risk of developing cancer and cardiovascular diseases (Pandey and Rizvi, 2009). Of note, the hypothesis that polyphenols constitute the active ingredients in food was supported by a wealth of *in vitro* and animal studies using isolated pure phenolic compounds (Gao and Hu, 2010). However, bioavailability studies in animal and human subjects have shown poor absorption and extensive hepatic metabolism of phenolic compounds, thus raising concerns on the applicability of *in vitro* studies in which parent phenolic compounds were used (Gao and Hu, 2010). In addition, these compounds were tested at concentrations (in μM) much higher than those achieved *in vivo* (in nM) (Gao and Hu, 2010). Thus, the following question arises: why are polyphenols bioactive despite low plasma concentrations? In order to answer this question, it should be taken into account that the dietary intake of polyphenols is estimated to reach values up to g quantities/day which is equivalent to mM levels of polyphenols and their metabolites in the gut (Grosso et al., 2017). It has thus been proposed that gut microbiota play a crucial rule in polyphenols' metabolism and activities (Williamson, 2013).

This section focuses on polyphenols-microbiota reciprocal interactions and their pivotal role in attenuating metabolic syndrome and type 2 diabetes mellitus.

#### Polyphenols as antidiabetic and anti-obesity agents

The antidibetic potential of polyphenols has been extensively studied and documented. *In vitro* studies show that polyphenols can inhibit the enzymes of dietary carbohydrate and lipid digestion such as α-glucosidase, α-amylase, and pancreatic lipase therefore reducing glucose and fatty acid intestinal absorption (Hanhineva et al., 2010). Several polyphenols such as quercetin, tea catechins, chlorogenic caffeic, and gallic acids were also reported to inhibit glucose absorption in intestinal Caco2 cell line as well as brush-border-membrane vesicles of porcine jejunum via the inhibition of sodium-dependent SGLT1 transporters (Hanhineva et al., 2010). GLUT2 was similarly inhibited by several flavonoids including quercetin, myricetin, neohesperidin, and catechin (Johnston et al., 2005; Kwon et al., 2007).

The insulin-responsive GLUT4, another glucose transporter responsible for glucose uptake in insulin-sensitive tissues, seems to be the target of many polyphenols. Some polyphenols stimulated GLUT4 translocation in adipocytes or skeletal muscle cells by activating either the insulin-mediated phosphatidylinositide 3-kinase (PI3-K)/Akt or the AMP-activated protein kinase (AMPK) pathways (Eid et al., 2015; Hajiaghaalipour et al., 2015). Regulation of AMPK activity can also leads to the activation of a class of protein deacetylases known as sirtuins. Activation of sirtuin 1 (Sirt1) is involved in the antiaging and anti-inflammatory effects of polyphenols such as resveratrol, querectin, catechins, and piceatannol (Chung et al., 2010). Polyphenols may also enhance glucose homeostasis by modulating hepatic glucose metabolism. Flavonoids such as tea catechins, quercetin and citrus flavonoids attenuated hepatic gluconeogensis in diabetic mice and rats through the inhibition of the key gluconeogenic enzymes glucose-6-phosphatase and phosphoenolpyruvate carboxykinase (Bahadoran et al., 2013; Eid and Haddad, 2017). Hepatic glucose kinase and glycogen synthase can also be modulated by polyphenols such as ferulic and hydroxylcinnamic acid derivatives (Bahadoran et al., 2013).

The molecular mechanisms of polyphenols' antidiabetic actions also include stimulation of insulin production and protection of pancreatic β cell against hyperglycemia-induced oxidative stress and promotion of β cell proliferation and survival in both *in vitro* and *in vivo* studies (Vinayagam and Xu, 2015).

Because of their unique chemical structures, polyphenols are powerful antioxidants. Green tea had the ability to scavenge 100% of superoxide anion and 86% of other reactive oxygen species (ROS) (Umeno et al., 2016). The radical scavenging properties of polyphenols were studied *in vitro* and were attributed to either modulation of enzymes responsible for the production of ROS including cyclooxygenase, lipoxygenase, xanthine oxidase, microsomal monooxygenase, and NADH oxidase (Bahadoran et al., 2013) or to the enhancement of the endogenous antioxidative defense system through the modulation of antioxidant enzymes like superoxide dismutase, catalase, and glutathione reducatse (Bahadoran et al., 2013). However, it is not clear if these *in vitro* results could be reproduced *in vivo* (Halliwell et al., 2005). In addition, when biomarkers of the antioxidant activities of polyphenols in animal and human subjects, including decreased LDL and DNA oxidation and increased plasma total antioxidant potential, were evaluated using fruit juices, soy or vegetables, it was found that the observed beneficial effect might not be attributed to polyphenols (Halliwell et al., 2005). The same authors proposed that the antioxidant activities of polyphenols could actually occur in the GI tract before absorption. They argued that, at best, polyphenol plasma concentrations do not exceed 1 μM, which are much lower than the concentrations they can reach in the gut, whereby the microbial fermentation products of polyphenols could be responsible for a greater proportion of antioxidant activities (Halliwell et al., 2005).

In addition to their antidiabetic properties, the antiobesity potential of some polyphenols is well documented in cell culture, animal and clinical studies (Wang et al., 2014). Resveratrol as well as green tea extract and its catechins, particularly epigallocatechin gallate (EGCG), were reported to suppress adipocyte differentiation in 3T3-L1 cell line through the activation of AMPK and the down regulation of adipogenic factors including peroxisome proliferator activator receptor γ (PPARγ) and CCAAT/enhancer binding protein α (C/EBPα) (Wang et al., 2014). Animal studies supported the effects of the aforementioned flavonoids on obesity-related inflammation and other obesity parameters such as body weight, total lipids, cholesterol and triglyceride (Khurana et al., 2013). In addition, enhanced fat oxidation in adipose tissue and skeletal muscle was reported in two obesity models: diet-induced obesity and ob/ob mice models treated with flavonoids (Khurana et al., 2013).

Clinical and epidemiological studies further confirmed the beneficial effects of certain polyphenols. A meta-analysis of 24 randomized clinical trials (RCTs) has documented the improvement of insulin resistance after daily consumption of flavonoid-rich cocoa beverages (Shrime et al., 2011; Ellinger and Stehle, 2016). Similarly, ingestion of chocolate, cacao or flavan-3-ol has significantly reduced insulin resistance and major cardiovascular diseases risk (CVD) factors in another meta-analysis of 42 RCTs (Hooper et al., 2008). Nevertheless, some other RCTs reported inconsistent results. For example, consumption of green tea containing 456 mg catechins (Fukino et al., 2005) or isoflavonoids in a 6-month RCT in postmenopausal Chinese women (Liu et al., 2010) did not prove beneficial regarding glucose levels or insulin resistance markers. Epidemiological studies also yielded conflicting results. A meta-analysis of 6 prospective cohort studies involving 284.806 participants has reported a significant inverse association between total polyphenols consumption and risk for type 2 diabetes in the US population (Liu et al., 2014). However, some inconsistent effects were found in other subgroups (Liu et al., 2014). Similar negative results were reported from other cross-sectional studies attempting to relate protection against diabetes to the intake of total flavonols and flavones (Song et al., 2005) or anthocyanins in the Iowa women (Nettleton et al., 2006). Inconsistent findings were also reported from RCTs investigating the antiobesity effects of flavonoids such as EGCG, resveratrol, and curcumin (Wang et al., 2014). Several factors might account for such inconclusive antidiabetic and anti-obesity clinical outcomes. Those include the duration of the study, age, gender, and ethnicity of participants or the varieties of polyphenols present in the diet.

#### Metabolism of polyphenols by gut microbiota

Initial bioavailability studies have reported poor absorption of flavonoids from food sources (Thilakarathna and Rupasinghe, 2013). Polyphenols are rarely found in nature in the free form. The majority of flavonoids monomer, except catechins, exist as β-glycosides (Marin et al., 2015). Catechins, being in the free form, are rapidly absorbed from the small intestine. On the other hand, the sugar moiety of the flavonoid glycosides determines their site of absorption. Hollman et al. (1995) detected the presence of quercetin glucoside in the circulation after ingestion of onion powder. Transport through SGLT1 was suggested as the mechanism of absorption (Hollman et al., 1995). Other studies suggested that glucosides could also be hydrolyzed by lactase phlorizin hydrolase (LPH), a β-glucosidase present in the intestinal brush border microvilli, to release the free aglycone. Absorbed agylcones mainly undergo hepatic phase II metabolism to form methyl, sulfate, and glucuronide conjugates (Marin et al., 2015). Other glycosides such as rhamnosides, arabinosides, and galactosides as well as some glucosides including cyanidin-3-glucoisde and the isoflavonoid daidzein-7-glucoside are not efficiently hydrolyzed by this enzyme and are moved to the colon where they are hydrolyzed and degraded by the colon microbiota (Marin et al., 2015).

Studies have reported that only 5–10% of dietary polyphenols can be absorbed from the small intestine. The remaining non absorbed (90–95%) polyphenols reach the colon at high concentrations (up to the mM range) where they undergo, together with the conjugates secreted in bile, deconjugation, and degradation by microbial enzymes to smaller phenolic compounds before being absorbed (Stevens and Maier, 2016). Oligomeric flavonoids are first degraded in the stomach to monomers and dimers while larger polymeric compounds such as ellagitannins are taken to the colon to be degraded by resident bacteria to their monomers (Kumar and Pandey, 2013).

Phenolic acids existing in free form, or conjugated to sugars, quinic, or shikimic acids, are absorbed from the small intestine after the hydrolysis of the conjugated forms. Importantly, the most abundant phenolic acids caffeic acid and ferulic acids are found generally bound to cell wall components such as lignins and polysaccharides and are therefore subject to colon microbial metabolism (Russell et al., 2009).

Gut microbes are not only capable of cleaving polyphenols glycosides and glucuronides, but they can also cleave the carbon-carbon bonds of heterocyclic and aromatic rings as well as decarboxylate, dehydoxylate, and hydrogenate alkene side chains (Stevens and Maier, 2016). The site of C-C cleavage seems to depend on the flavonoid subclass: in anthocyanidins, flavonols, flavones, and flavanones, the cleavage occurs in ring-C, while in flavanols, it also takes place in ring-A (Figure 3).

Phenolic acids are the major metabolites of flavonoids detected in circulation and urine (Stevens and Maier, 2016). Ring-C cleavage of anthocyanins produces many phenolic acids such protocatechuic (PCA), gallic, 3-O-methyl-gallic, syringic, p-coumaric, and vanillic acids, as well as 2,4,6-trihydroxybenzaldehyde (Duda-Chodak et al., 2015). Interestingly, many biological activities have been attributed to PCA such as antioxidant, anti-inflammatory, antihyperglycemic, antiatherogenic, hypocholeserolemic, anticancer, and neuroprotective actions (Duda-Chodak et al., 2015). In addition, anthocyanin metabolites, namely gallic acid, 3-O-methyl-gallic acid, and 2,4,6-trihydroxybenzaldehyde, were more effective as cytotoxic compounds than the parent anthocyanin (Forester and Waterhouse, 2010). Similarly, flavonols such as quercetin are metabolized to PCA and 2-(2,4,6-trihyrdoxyphenyl)-acetic acid, which can be further converted to their o-methyl conjugates. Flavanols such as catechin, epicatechin, and ECGC undergo both A-ring and C-ring cleavage to form 3-hydroxyphenylacetic acid, 3-hydroxyphenylpropionic acid, 3,4-dihydroxyphenylacetic acid, and 3-hydroxyphenyl-γ-valerolactone. Demethylation and ring-C fission of the isoflavonoid diadezein generates the major metabolite O-demethyl-langlensin (Stevens and Maier, 2016). Ellagitannins are degraded to ellagic acid, which is further metabolized by the colon microflora to the phenolic metabolites called urolithins. Phenolic acids such as caffeic acid and its ester chlorogenic acid are metabolized by the microflora to phenylpropionic, benzoic and, hippuric acids (Gonthier et al., 2003). Caffeic acid can also undergo decarboxylation and transformation by gut microbiota into 4-ethyl catechol (Ozdal et al., 2016). Lignans such as pinoresinol and syringaresinol are biotransformed to enterolactone and enterodiol, two phtoestrogens responsible for estrogenic agonistic/antagonistic activities of lignans (Ozdal et al., 2016). Finally, microbial communities also possess glucuronidases and sulphatases, thus transforming phase II metabolites into the free form and enabling their absorption into the general circulation (Cardona et al., 2013).

The biological activities of the microbial metabolites of polyphenols are still under investigation. However, because they are efficiently absorbed by the GI tract, they are believed to be responsible for the health promoting activities of polyphenols (Cardona et al., 2013). In a recent study, the two metabolites 3-hydroxyhippuric acid and 3-hydroxyphenyl acetic acid were found to reach and accumulate in the brain of rats treated with grape seed phenol extract in a dose dependent manner (Wang D. et al., 2015).

#### Modulation of microbiota by polyphenols

Because of their distinctive structures, polyphenols have shown a great potential as antimicrobial agents against human pathogens in experimental and clinical studies (Coppo and Marchese, 2014). Their activities against gastrointestinal tract pathogens (Selma et al., 2009) are of particular interest to this review. Commensal and probiotic microorganisms such as strains of *Lactobacillus, Streptoccoccus, Bifidobacteria*, and the yeast *Saccharomyces boulardii* are known to preserve epithelial integrity, protect against enteric pathogen, and serve other important function such as modulating sugar and lipid metabolism. The gut can also be inhabited by pathogenic bacteria that cause GI illness such as inflammatory bowel disease caused by *Clostridium difficile* infection (Bien et al., 2013). Dysbiosis is the term that describes the imbalance between the beneficial and pathogenic bacteria. It has been associated with many chronic diseases including metabolic disorders such as obesity and type 2 diabetes (Carding et al., 2015). Since Firmicutes possess fewer enzymes than Bacteriodetes capable of degrading glycans, products of polyphenol glycoside hydrolysis, it was suggested that the intake of polyphenols could influence the composition of gut microbiota in favor of beneficial bacteria with health promoting functions (Cardona et al., 2013). Thus, it is deemed necessary to investigate the effect of polyphenols on human gut bacterial growth.

The modulation of gut microbiota by polyphenols was firstly studied *in vitro*. One study examined the effect of some flavonoids aglycones and their glycosides on representative species of human gut microbial communities (*Bacteroides galacturonicus, Lactobacillus* sp., *Enterococcus coccae, Bifidobacterium catenulatum, Ruminococcus gauvreauii, E. coli*). The aglycones quercetin, naringenin and hesperetin but not their glycosides rutin, naringin and hesperidin inhibited the growth of certain tested bacteria (Duda-Chodak et al., 2015). In the same study, catechin had no impact on the growth of the aforementioned bacteria (Duda-Chodak et al., 2015). In another *in vitro* study, the flavonol galangin but not quercetin or fisetin inhibited the growth of *B. adolescens* (Kawabata et al., 2013). Similarly, the effects of the common dietary polyphenols on the growth and adhesion of commensal and pathogenic bacteria to enterocytes were studied in the Caco2 cell line (Parkar et al., 2008). At their physiological concentration, all polyphenols, except rutin, affected the viability of representative gut flora. However, one of the major limitations of *in vitro* studies is that 80% of the bacteria in the human gut are uncultured. Interestingly, incubation of anthocyanins with fecal bacteria in stirred, batch-culture fermentation vessels simulating human colon conditions resulted in an increase of the beneficial *Bifidobacterium* spp. and *Lactobacillus-Enterococcus* spp. levels (Hidalgo et al., 2012).

More interesting results have emerged from *in vivo* and clinical studies. Ellagitannin-rich pomegranate extract (POME) and ellagitannins main metabolite urolithin-A were reported to significantly enhance the growth of *Bifidobacterium* and *Lactobacillus* spp. in a rat model of colitis. Interestingly, urolithin-A has shown more powerful anti-inflammatory properties than the parent ellagitannin-rich extract (Larrosa et al., 2010). In a clinical study, the consumption of 1,000 mg of POME by 20 healthy volunteers for 4 weeks resulted in beneficial changes in the gut microbiota of participants who were able to metabolize ellagitannins to urolithin-A. These changes include reduction in *Firmicutes* levels and the induction of the growth of *Enterobacter, Lactobacillus, E. coli*, and *Verrucomicrobia* (*A. muciphila*). The latter two bacterial strains were reported to preserve the intestinal mucus and the glycocalyx layer and to reduce the intestinal permeability frequently associated with development of inflammation and metabolic diseases (Zhang and Zhang, 2013).

Of note, administration of both quercetin and resveratrol (Etxeberria et al., 2015), or resveratrol alone (Qiao et al., 2014), to obese rat fed high fat/high sucrose (HFHS) or high fat diets was reported to reduce F/B ratio (Qiao et al., 2014; Etxeberria et al., 2015). Moreover, it decreased the abundance of bacteria associated with diet-induced obesity, such as *Bacillus* spp., *Eubacterium cylindroide*, and *Erysipelotrichaceae* (Etxeberria et al., 2015) and increased the growth of *Bifidobacterium* and *Lactobacillus* (Qiao et al., 2014; Etxeberria et al., 2015).

The highly polymeric apple procyanidins administered for 20 weeks to C57BL/6J mice fed a HFHS diet attenuated weight gain and caused a reduction in inflammation and gut permeability, as well as modulation of lipid metabolizing genes (Masumoto et al., 2016). Similarly, apple peal polyphenols upregulated antioxidant enzymes and attenuated experimental inflammatory bowel disease (Denis et al., 2016). Furthermore, F/B proportion was markedly decreased and *Akkermansia* levels were increased eight-fold by the end of the treatment (Masumoto et al., 2016). Grape/red wine polyphenols (Roopchand et al., 2015) and procyanidin-rich cranberry extract (Anhe et al., 2015a) showed similar effects on obesity, metabolic syndrome parameters and levels of *Akkermansia* in mice fed a high-fat diet (Anhe et al., 2015a; Roopchand et al., 2015).

Collectively, these studies reveal that polyphenols act as prebiotic supplements that can positively modulate gut microbiota composition mainly through the enrichment of beneficial bacteria and the inhibition of pathogenic bacterial growth (Anhe et al., 2015b; Le Barz et al., 2015; Fändriks, 2017).

### Polysaccharides

Carbohydrates are classified based on the number of linked carbohydrate units into mono-, oligo-, and poly-saccharides (Cummings and Stephen, 2007). For instance, glucose, fructose, and galactose are monosaccharides whereas lactose, sucrose, and maltose are disaccharides. Common oligosaccharides include fructo-oligosaccharides (FOS), galacto-oligosaccharides (GOS), and mannan oligosaccharides (MOS). Arabinoxylans, cellulose, chitin, and pectins are examples of polysaccharides having linear or branched structures (Cummings and Stephen, 2007).

Another classification is based on whether carbohydrates are digestible and non-digestible. Starch, dextrin, glycogen are some of the digestible polysaccharides, while non-digestible carbohydrates (NDCs) (Paeschke and Aimutis, 2010) are listed in Table 7 (Kaushik et al., 1989).

**Table 7:** Examples of NDCs.

**Class**	**Example**
Fructans	Inulin, FOS, oligofructose (OFS), levan
Galactans	(Galactooligosaccharides) GOS, trans-galactooligosaccharides (TOS)
Grains, fruits, vegetables-oligo and polysaccharides	Pectin, β-glucan, cellulose, hemicellulose, arabinoxylan, arabinoxylooligosaccharides (AXOS)
Synthetic NDCs	Polydextrose, resistant maltodextrin
Resistant starches (Type 1 to 4)	
Galactomannan polysaccharides	Gums of guar, hydrolyzed guar, locust bean, genugreek, tara
Microbial polysaccharides	Xanthan and gellan gum
Seaweed polysaccharides	Alginate
Glucomannan polysaccharides	Konjac
Tree exudate polysaccharides	Gum arabic, gum acacia, karaya, tragacanth and ghatti gums

#### Gut microbiota and dietary intake

Some of the gut's microbial species are infulenced by the composition of the diet (Kok et al., 1996; Wu et al., 2011; Pyra et al., 2012). Firmicutes and Proteobacteria (Prevotella enterotype) thrive under diets that are rich in carbohydrates and simple sugars (such as glucose and fructose), while Bacteroidetes and Actinobacteria (Bacteroides enterotype) are favored by diets rich in saturated fat and animal protein. Fat and protein decrease the microbial diversity of the intestine, whereas carbohydrates increase it (Wu et al., 2011). Oligo- and poly-saccharides enhance the concentration of the species belonging to Actinobacteria and Firmicutes phyla except for Staphylococcus (*Staphylococcus aureus*), Clostridium (*C. difficile, Clostridium leptum, Clostridium perfringens*). Proteobacteria phyla show the opposite trend, whereas Bacteroidetes and Verrucomicrobia show a mixed trend (Wu et al., 2011).

#### Actions of oligo- and poly-saccharides

Oligo- and poly-saccharides exert a significant influence on the gut microbiota. In general, oligo- and poly-saccharides increase short-chain fatty acids (SCFA), GLP-1, and PYY, while decreasing triglycerides, VLDL, and LDL, with a mixed trend being observed for HDL (Morrison and Preston, 2016), as shown in Table 8. These changes and their metabolic consequences will be summarized below.

**Table 8:** Influence of oligo- and poly-saccharides on key metabolic regulators.

	**SCFA**	**TG**	**VLDL**	**GLP-1**	**HDL**	**PYY**	**LDL**
Inulin	Increases (Woods and Gorbach, 2001)	Decreases (Russo et al., 2008)	Decreases (Delzenne et al., 2002; Koleva et al., 2012; Mao et al., 2015; Hashmi et al., 2016; Liu T. W. et al., 2016)				
FOS	Increases (Woods and Gorbach, 2001)	Decreases (Modler, 1994)		Increases (Piche et al., 2003; Hong et al., 2016)			
GOS	Increases (Woods and Gorbach, 2001)	Decreases (Hashmi et al., 2016)	Decreases (Hashmi et al., 2016)	Increases (Hong et al., 2016)	Increases (Kok et al., 1996; Pyra et al., 2012; Hashmi et al., 2016)	Increases (Hong et al., 2016)	Decreases (Hashmi et al., 2016)
OFS		Decreases (Daubioul et al., 2002)	Decreases (Delzenne et al., 2002; Russo et al., 2008)	Increases (Delzenne et al., 2005)		Increases (Delzenne et al., 2005)	
ITF		Decreases (Backhed et al., 2004)		Increases (Delzenne et al., 2007; Koleva et al., 2012; Mao et al., 2015; Liu T. W. et al., 2016)	Decreases (Kok et al., 1996; Rajpal et al., 2015; Liu F. et al., 2016)		Decreases (Liu F. et al., 2016)
Fructans	Increases (Clarke et al., 2016)	Decreases (Marquez-Aguirre et al., 2016)		Increases (Parnell and Reimer, 2009)	Increases (Rendon-Huerta et al., 2012)	Increases (Parnell and Reimer, 2009)	Decreases (Bindels et al., 2012; Rendon-Huerta et al., 2012; Chan et al., 2016)
β-glucans		Decreases (Hong et al., 2005; Riviere et al., 2016)			Increases (Hong et al., 2005)		
AXs	Increases (Van den Abbeele et al., 2011)	Decreases (Adam et al., 2001)					
AXOS	Increases (Backhed et al., 2004; Delzenne et al., 2007; Wu et al., 2010; Geraylou et al., 2013)			Increases (Malaguarnera et al., 2012; Neyrinck et al., 2012; Salazar et al., 2015; Liu F. et al., 2016)		Increases (Neyrinck et al., 2012)	
XOS		Decreases (Modler, 1994; Hsu et al., 2004; Salazar et al., 2015)					
PDX		Decreases [68]		Increases (Olli et al., 2015)			
RMD				Increases (Hira et al., 2015)			
RS	Increases (Hu et al., 2016)	Decreases (Bronkowska et al., 2013)		Increases (Keenan et al., 2015)			
Guar gum		Decreases (den Besten et al., 2014)		Increases (Williams, 1999; Adam and Westerterp-Plantenga, 2005)			

Firstly, the fermentation of non-digestible carbohydrates takes place in the gut and produces SCFAs, with acetate, propionate, and butyrate (Morrison and Preston, 2016) present in a ratio of 3:1:1 (Hoverstad et al., 1984). More specifically, *A. municiphilla* produce propionates (Derrien et al., 2004) while *Ruminococcus bromii* (Ze et al., 2012) *F. prausnitzii, Eubacterium rectale, Eubacterium hallii, and Ruminococcus bromii*, produce butyrate (Louis et al., 2010). Propionates and butyrates lower lipogenesis and serum cholesterol level while activating intestinal gluconeogenesis (IGN) which contributes to glucose homeostasis (Hosseini et al., 2011).

SCFA are ligands of the free fatty acid receptor (FFAR) 2 and 3, also known as G-protein coupled receptor (GPR) 43 and 41, respectively. FFAR2 is activated by acetate and propionate, while FFAR3 is activated by propionate and butyrate (Kaji et al., 2014). Propionate is involved in gluconeogenic activity while acetate and butyrate contribute to lipogenic activity (den Besten et al., 2013a). These three gut bacterial products (acetate, propionate, and butyrate) thus regulate gluconeogenesis and lipogenesis, thereby modulating hepatic lipid and glucose homeostasis, notably through PPAR-γ (den Besten et al., 2015). SCFAs produced from dietary fibers can also increase LPS (Blaut and Klaus, 2012), the main component of gram-negative bacteria (Delzenne and Cani, 2011). Plasma LPS participates in the development of insulin resistance, as discussed by Carvalho et al. (2012). In contrast to SCFAs, high concentrations of omega-3 fatty acids reduce LPS (Kaliannan et al., 2015), as do Gram-positive bacteria (Bifidobacterium and Lactobacillus) (Zhang et al., 2010).

On the other hand, the activity of gut microbiota influences triglycerides, which represent stored glycerol-linked fatty acids (den Besten et al., 2013b). Different concentrations of cholesterol, lipoproteins, and triglycerides determine the classification of circulating lipids into chylomicrons, very low-density lipoproteins (VLDL), intermediate-density lipoproteins (IDL), low-density lipoproteins (LDL), and high-density lipoproteins (HDL) (Cox and Garcia-Palmieri, 1990). It is well-established that diets rich in fructose can increase triglycerides (Schaefer et al., 2009). High triglyceride levels, in turn, bring out undesirable effects on lipid metabolism (Reiser, 1985) and, as detailed above, are one of the hallmarks of the metabolic syndrome. In contrast, decreased triglyceride levels reduce fatty acid synthase activity (Morand et al., 1994) and provide beneficial health effects. Several polysaccharides such as inulin, FOS, GOS, OFS, ITF, fructans, β-glucans, arabinoxylans, xylo-oligosaccharides, polydextrose, resistant starch, and guar gum decrease triglyceride levels (de Deckere et al., 1993).

Dietary carbohydrates also modulate important incretin hormones, namely glucose-dependent insulinotropic peptide (GIP) and glucagon-like peptide-1 (GLP-1) (Edholm et al., 2010). Endocrine K-cells secrete GIP in response to carbohydrates and fat ingestion, (Andersen et al., 1978) whereas, GLP-1 is secreted by L cells (Kieffer and Habener, 1999) in response to luminal sugars, amino acids and fatty acids (Holst, 2007). GLP-1 stimulates the release of insulin, suppresses the release of glucagon (Nadkarni et al., 2014), lowers the blood glucose (Nauck et al., 1993), and plays a major role in glucose homeostasis (Kieffer and Habener, 1999; Deacon, 2004). GIP exerts similar effects (Yabe and Seino, 2011). Oligo- and poly-saccharides such as fructo-oligosaccharides (FOS), Glucooligosaccharides (GOS), oligofructoses (OFS), inulin-type fructans (ITF), fructans, Arabinoxylooligosaccharides (AXOS), resistant maltodextrin, resistant starch, and guar gum increase GLP-1 (Wang X. et al., 2015).

Another important peptide hormone modulated by dietary carbohydrates is *peptide* YY (PYY), which is secreted by α-cells of the pancreatic islets in response to the food that is ingested (Shi et al., 2015). External administration of SCFAs releases PYY and GLP-1 into plasma (Freeland and Wolever, 2010), while generated SCFAs can increase PYY concentration only (Cherbut et al., 1998). When PYY concentration increases, serum insulin level also increases, which improves glucose tolerance (Shi et al., 2015) leading to GH regulation (Boey et al., 2007). Therefore, the higher level of GLP-1, GIP, and PYY is desirable.

Gut microbiota modulates the brown adipose tissues (BATs) (Mestdagh et al., 2012). When stimulated, FFA are used-up to clear the TGs (Bartelt et al., 2011) in turn regulating glucose homeostasis (Stanford et al., 2013) (Peirce and Vidal-Puig, 2013).

Sodium-glucose transport protein-1 (SGLT-1) and glucose transporter-2 (GLUT-2) also participate in maintaining GH (Kellett and Helliwell, 2000).

## Summary

The healthy human gut represents a complex and highly variable ecological system consisting of several microbes belonging to bacteria, fungi and virus domains, in addition to host epithelial cells. Firmicutes, Bacteroidetes, Proteobacteria, Actinobacteria, and Fusobacteria are the major phyla colonizing the stomach and intestine of healthy adults. The exact role of gut microbiota is not fully elucidated but many studies implicate microbiota to perform tasks that are known to be useful for the human host such as modulating intermediate metabolism and the immune system. Diet-induced changes in the composition/diversity of gut microbes are thus believed to participate in the pathogenesis of certain diseases through modifying different metabolic processes in the host.

Less diverse intestinal microbiota have been reported in metabolic disorders. While the association between increased Firmicutes/Bacteroidetes ratio and metabolic diseases is still controversial, more recent studies associated *Akkermansia* and *Lactobacillus* species with central obesity and fasting hyperglycemia.

Polyphenols, oligo-, and poly-saccharides can influence the composition of gut microbiota by favoring beneficial bacteria and inhibiting growth and activity of pathogenic species and thus constitute a promising avenue for the prevention and treatment of metabolic disorders.

## Future challenges and opportunities

Given all the above considerations, the perspectives for targeting the gut microbiome in the context of metabolic diseases keeps being highly relevant and timely. First and foremost, there remains a need to refine research on specific microbes that may be more specifically involved in metabolic diseases rather than considering broader categories, such as phyla. Among challenges that should be met, more studies should focus on the roles and potential mechanisms of action of non-bacterial gut microbes, since these remain poorly understood. Continued research efforts should also result in the better understanding of the modes of action of pre- and pro-biotics in metabolic diseases, notably in terms of metabolic and inflammatory mediators.

There also remains a lot to be done to further elucidate the intricate interactions between prebiotics (polyphenols and fibers) on the one hand, and probiotics (gut microbes), on the other, notably in what pertains to the metabolism of prebiotics by the latter and the influence this has on the bioactivity of the former. In this context, experimental approaches and tools have now evolved that can meet this challenge. For instance, one could think of combining bacterial metagenomics with plant metabolomics and hence study relationships through the use of powerful bioinformatics.

Overall, and in a very pragmatic sense, academics and industrial partners will need to work together to develop safe and reliable products that can help prevent and mitigate the ill effects of metabolic syndrome and related obesity and diabetes. In this context, a promising approach may be to further explore symbiotic products that can combine pre- and pro-biotics in novel and efficient ways.

## Author contributions

HE, MW, NA, AQ, AA, and PH have written the first draft of the manuscript. SH, AM, SN, and LR revised and improved the first draft. All authors have seen and agreed on the final submitted version of the manuscript.

### Conflict of interest statement

The authors declare that the research was conducted in the absence of any commercial or financial relationships that could be construed as a potential conflict of interest.
